# Dual Vacancy Engineering in Alloyed Ga‐Zn‐Cu‐Se Quantum Dots for Photocatalytic 5‐Hydroxymethylfurfural to 2,5‐Diformylfuran Conversion

**DOI:** 10.1002/adma.202510164

**Published:** 2025-08-27

**Authors:** Meijun Guo, Tianyu Zhao, Shuangming Chen, Li Song, Bingquan Xia, Jingrun Ran, Shi‐Zhang Qiao

**Affiliations:** ^1^ School of Chemical Engineering The University of Adelaide Adelaide SA 5005 Australia; ^2^ School of Chemistry and Chemical Engineering Harbin Institute of Technology Harbin 150001 P. R. China; ^3^ National Synchrotron Radiation Laboratory CAS Centre for Excellence in Nanoscience University of Science and Technology of China Hefei 230029 P. R. China; ^4^ Key Laboratory for Green Chemical Process of Ministry of Education School of Chemistry and Environmental Engineering Wuhan Institute of Technology Wuhan 430074 P. R. China

**Keywords:** biomass conversion, dual vacancy engineering, Ga‐Zn‐Cu‐Se quantum dots, in situ characterizations, photocatalysis

## Abstract

Highly ‐active/‐selective photocatalytic biomass conversion is of great importance for achieving remarkable solar‐to‐chemical conversion. However, serious challenges, e.g., limited photon utilization, high charge recombination, and sluggish/uncontrolled reaction kinetics, remain for further development in this area. Herein, a dual‐vacancy‐engineering strategy is employed to regulate the Ga‐Zn co‐doped Ga‐Zn‐Cu‐Se (GZC) quantum dots (QDs) by a cation exchange route utilizing the CuSe template. The optimized GZC QDs exhibit excellent photocatalytic performances for the selective oxidation of 5‐hydroxymethylfurfural (HMF) into 2,5‐diformylfuran (DFF), with 89% HMF conversion and 91% DFF selectivity. Advanced ex situ/in situ characterizations, together with theoretical calculations, reveal the origins of the excellent performance: i) Zn doping enhances charge carrier mobility, thereby promoting the HMF‐to‐DFF conversion rate; ii) Ga doping introduces intermediate states in electronic structure, facilitating better charge separation/transfer; iii) Ga/Zn co‐introduction results in formation of Se/Cu vacancies, which play a critical role in charge separation and reactive oxygen species generation. Overall, the research exhibits a rational strategy for designing vacancy‐engineered photocatalysts, offering a promising approach for selective biomass conversion.

## Graphical abstract

A dual‐vacancy‐engineered strategy is developed to construct Ga‐Zn co‐doped CuSe quantum dots (GZC QDs) via cation exchange using CuSe as a template. By precisely introducing Cu and Se vacancies, the optimized sample efficiently facilitates the selective photocatalytic oxidation of 5‐hydroxymethylfurfural (HMF) to 2,5‐diformylfuran (DFF), achieving an HMF conversion rate of 89% and a DFF selectivity of up to 91%.

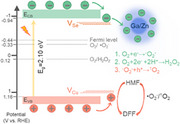

## 1 Introduction

Semiconductor photocatalysis offers a promising route to address both energy and environmental challenges while enabling the generation of valuable chemical products.^[^ ^1^, ^2^ ^]^ For effective utilization of solar energy through photocatalytic processes, it is essential to select materials that not only possess suitable light absorption properties but are also sustainable and cost‐effective.^[^ ^3^ ^]^ Cu‐based chalcogenide (CuSe and CuS) quantum dots (QDs), composed of earth‐abundant elements with optimal bandgaps allowing broad spectra response, are promising candidates for photocatalytic applications.^[^ ^4^, ^5^ ^]^ However, the quick recombination of photo‐generated electron‐hole pairs in single‐component nanocrystals greatly limits the efficiency of photocatalytic reactions powered by solar energy. While constructing heterojunctions is an extensively adopted strategy to enhance photoelectric performance, mismatched lattice structure‐induced defects at the interface are challenging to control, which in turn acts as trapping sites for charge carriers.^[^ ^6^, ^7^ ^]^ Constructing ternary or quaternary chalcogenide QDs via cation exchange process offers an atomically uniform structure and electronically advantageous alternatives, which preserves lattice continuity, minimizing interfacial defects and enhancing charge mobility.^[^ ^8^, ^9^, ^10^ ^]^ Both CuSe and CuS materials are ideal templates for cation exchange owing to their intrinsic Cu vacancies, structural anisotropy, and favorable exchange kinetics, resulting in efficient and morphology‐preserving transformations in mild conditions.^[^ ^11^, ^12^ ^]^ For example, Gao et al. achieved site‐selective Fe^2+^ incorporation into ZnS‐coated CdSe QDs via cation exchange, remarkably raising photocatalytic H_2_ evolution and durability.^[^ ^13^ ^]^ Similarly, Liu et al. reported the rapid conversion of CuInS_2_ into biconcave Cu_1.94_S nanoplates through In^3+^ extraction, generating abundant defects during the phase transition.^[^ ^14^, ^15^ ^]^ These regulations not only introduce vacancies, but also result in optimized band structure and raised visible‐light absorption, thus promoting catalytic activity. These features render Cu‐based materials promising platforms for efficient/selective solar‐to‐chemical conversion, because of rapid charge separation and increased active sites. Despite some progress in this field, atomic‐level modulation of quaternary Cu‐based chalcogenides through dual‐metal co‐doping and the associated dual‐vacancy engineering remains largely unexplored.

The catalytic transformation of renewable and plentiful lignocellulosic biomass into value‐added chemicals offers a promising pathway toward achieving carbon neutrality. Among biomass‐derived platform molecules, 5‐hydroxymethylfurfural (HMF) has been recognized by the U.S. Department of Energy as one of the top 12 renewable feedstock chemicals, due to its potential to be converted into a wide range of high‐value products.^[^ ^16^, ^17^ ^]^ One of the most important derivatives for advancing bio‐based material development of HMF is 2,5‐diformylfuran (DFF), which is a key resource to produce furan‐based polymers, pharmaceutical intermediates, fungicides, etc.^[^ ^18^, ^19^ ^]^ The photocatalytic selective‐oxidation technique has emerged as a green alternative for realizing HMF‐to‐DFF conversion, but its application remains constrained by the weak visible‐light absorption, rapid charge recombination, and insufficient active sites of current photocatalysts.

Herein, our report for the first time introduces an innovative route for preparing alloyed quaternary Ga‐Zn‐Cu‐Se (GZC) quantum dots (QDs) with small sizes of 6‐8 nm, via a facile cation exchange route utilizing CuSe as a template. The well‐designed GZC QDs (GZC‐2) exhibit remarkable photocatalytic efficiency for HMF‐to‐DFF conversion, achieving 89% HMF conversion and 91% DFF selectivity. This performance is over 90‐fold higher than that of CuSe. The X‐ray photoelectron spectroscopy (XPS), electron paramagnetic resonance (EPR) spectroscopy, and X‐ray absorption near‐edge structure (XANES) spectra together reveal that with the co‐doping of Zn and Ga, the introduction of Se and Cu vacancies (labelled as V_Se_ and V_Cu_) is also realized. V_Se_ and V_Cu_ serve as active sites that regulate electron transfer and reactive oxygen species generation, resulting in superior selectivity for the photocatalytic selective oxidation of HMF into DFF. These are supported by the in situ EPR technique. Furthermore, theoretical calculations, transient absorption spectroscopy (TAS), transient‐state photoluminescence (PL) spectroscopy, and transient‐state surface photovoltage (TPV) spectroscopy demonstrate that these vacancy‐induced mid‐gap states significantly facilitate charge separation/transfer in GZC‐2. All the above results highlight the key role of atomic‐level dual vacancy engineering in designing/preparing high‐performance photocatalysts for biomass conversion.

## 2 Results and Discussion

### 2.1 Composition and Structure of Regulated Ga‐Zn‐Cu‐Se Quantum Dots

The incorporation of Zn and Ga into CuSe quantum dots (QDs) enables bandgap tuning and promotes the formation of vacancy defects, rendering them promising candidates for photocatalytic selective oxidation of 5‐hydroxymethylfurfural (HMF) into 2,5‐diformylfuran (DFF). The preparation of alloyed quaternary Ga‐Zn‐Cu‐Se (GZC) QDs requires the application of a two‐step hot‐injection route, as explained in the supporting information. The first hot‐injection route results in the preparation of CuSe QDs with a packing arrangement as the initial template.^[^ ^20^ ^]^ Furthermore, to introduce Ga and Zn into the CuSe host structure via a cation exchange process, the as‐prepared CuSe QDs suspension was injected into a hot solution containing Ga and Zn precursors. The suspension was stirred at 150 °C for 5, 10, and 15 min, resulting in catalysts labelled as GZC‐1, GZC‐2, and GZC‐3, respectively. As a control, Zn‐Cu‐Se (ZC) QDs were prepared under identical conditions except in the absence of the Ga precursor, with a 10‐min reaction time. And Ga‐Cu‐Se (GC) QDs were also prepared under the identical conditions except in the absence of Zn precursor, with a 10‐min reaction time. All the preparation details can be found in the supporting information.

**Figure 1:**
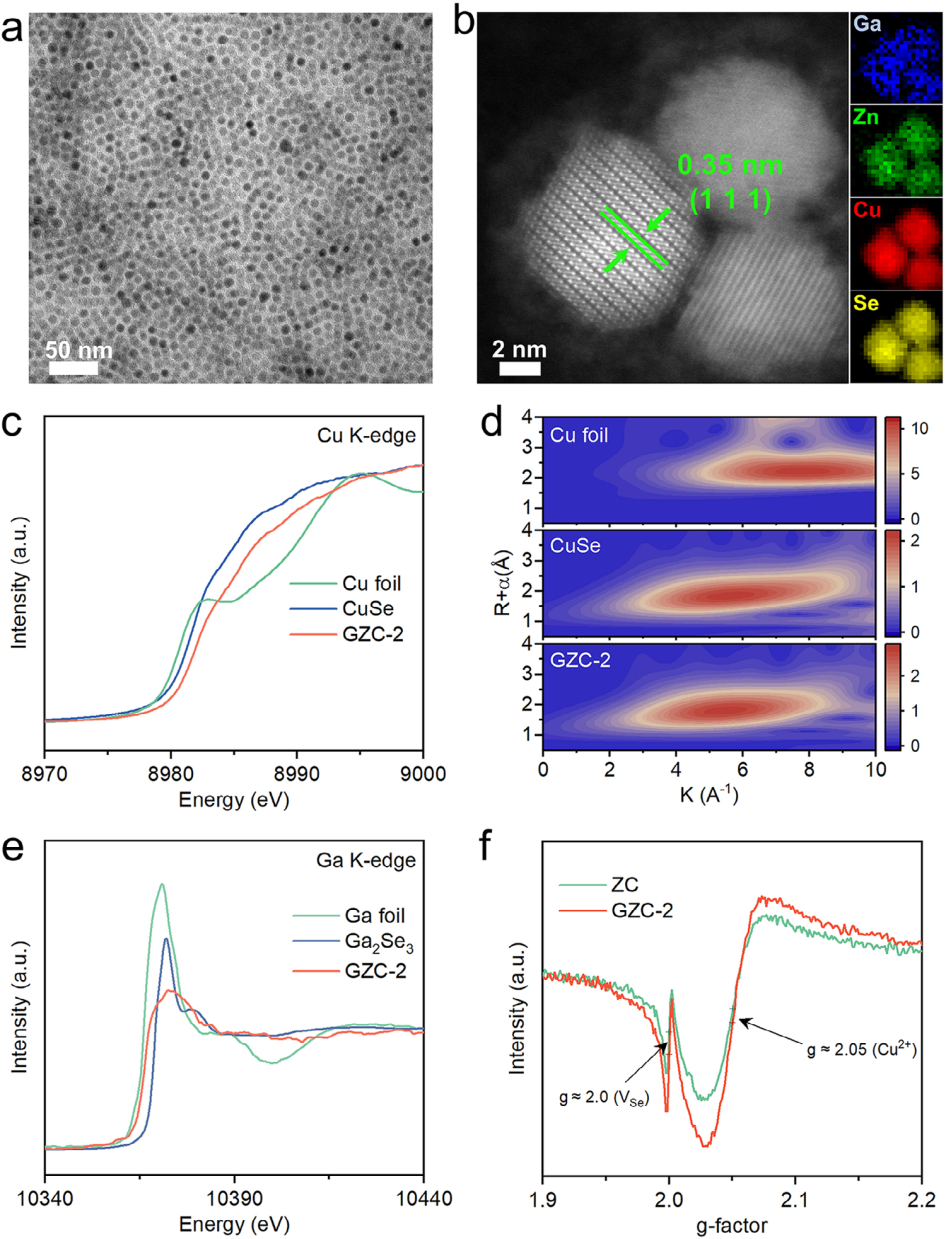
a) TEM image and b) HAADF‐STEM image of GZC‐2 QDs with corresponding EELS elemental mapping images for Ga, Zn, Cu, and Se, respectively. c) Cu K‐edge XANES spectra and d) WT contour plots of Cu K‐edge EXAFS spectra for Cu foil, CuSe, and GZC‐2. e) Ga K‐edge XANES spectra for Ga foil, Ga_2_Se_3_, and GZC‐2. f) EPR spectra for ZC and GZC‐2.

The microstructural features of the as‐prepared CuSe and GZC‐2 QDs were examined by transmission electron microscopy (TEM). As exhibited in Figure S1a (Supporting Information), the CuSe QDs exhibit a uniform particle size distribution and serve as the template for subsequent cation exchange. The high‐angle annular dark‐field scanning transmission electron microscopy (HAADF‐STEM) image (Figure S1b, Supporting Information) presents well‐defined lattice fringes with a spacing of 0.36 nm, corresponding to the (102) plane of Cu_0.87_Se. Elemental mapping images via electron energy loss spectroscopy (EELS; Figure S1c, Supporting Information) confirm the uniform distribution of Cu and Se, further validating the composition of CuSe QDs. Additionally, particle size analysis (Figure S2, Supporting Information) exhibits that most CuSe QDs fall within the 6‐8 nm range. X‐ray diffraction (XRD) analysis (Figure S3, Supporting Information) confirms that the as‐prepared CuSe QDs possess a crystal structure consistent with klockmannite Cu_0.87_Se (PDF #83‐1814). This non‐stoichiometric phase, featured by intrinsic Cu vacancies, facilitates cation exchange by providing accessible migration sites for Zn and Ga, without substantially disrupting the host lattice.^[^ ^21^ ^]^ Following the cation exchange process, the morphology and size of GZC‐2 QDs (**Figure** **1**a) are preserved, indicating the structural integrity of the CuSe template. The HAADF‐STEM image (Figure 1b) reveals well‐defined lattice fringes with an interplanar distance of ≈0.35 nm, corresponding to the (111) facet of the cubic zinc blende phase of ZnSe. This result reveals a lattice‐preserving transformation upon multi‐cation incorporation.^[^ ^22^ ^]^ Particle size analysis (Figure S4, Supporting Information) presents that GZC‐2 QDs maintain a size distribution of 6‐8 nm, consistent with that of CuSe (Figure S2, Supporting Information). This result further confirms the morphological stability during exchange. Elemental mapping images (Figure 1b inset) reveal a homogeneous distribution of Zn, Ga, Se, and residual Cu, with no evidence of phase separation. These results reveal the substitutional doping mechanism, forming a multi‐cationic solid solution. Such uniform incorporation of dopants is expected to promote the formation of spatially distributed active sites and enhance charge separation/transport, potentially improving photocatalytic performance. The XRD patterns of GZC‐1, GZC‐2, and GZC‐3 QDs (Figure S5, Supporting Information) correspond to cation exchange durations of 5, 10, and 15 min, respectively. The results reveal a gradual movement of diffraction peaks toward higher angles for GZC‐2 and GZC‐3, compared to those of GZC‐1. The corresponding atomic ratios of Ga: Zn: Cu in various GZC QDs were determined by inductively coupled plasma‐atomic emission spectroscopy (ICP‐AES). The results are exhibited in Table S1 (Supporting Information), which were calculated by normalizing the Cu content to 1. These results reveal the gradual increase of both Ga and Zn contents with elongated cation exchange times for GZC‐1 (5 min), GZC‐2 (10 min), and GZC‐3 (15 min). This trend also corroborates the successful incorporation of more Ga and Zn into the CuSe lattice with extended exchange time. The XRD peaks of GZC‐2 and GZC‐3 are approaching the characteristic peaks of the cubic phase ZnSe (PDF #88‐2345). These results indicate a reduction in lattice parameters, owing to the structural transition from hexagonal CuSe (klockmannite) to cubic zinc blende upon Ga and Zn incorporation. As exhibited in Figure S3 (Supporting Information), under identical reaction conditions, the peak positions of GZC‐2 and ZC QDs exhibit a negligible shift. This can be attributed to the low Ga doping level in GZC‐2 and the comparable ionic radii of Ga^3+^ and Zn^2+^, which exerts minimal influence on the crystal lattice.^[^ ^23^ ^]^ Thus, the incorporation of Ga alongside Zn does not significantly alter the overall crystal structure. To obtain clean powders for subsequent characterization, the synthesized samples were fully washed with n‐hexane and methyl acetate, respectively, to remove residual organic ligands. Successful ligand removal was confirmed by the FTIR spectra (Figure S6, Supporting Information). As exhibited in Figure S6 (Supporting Information), the oleylamine‐related signals (e.g., the C‐H stretching vibrations at ≈2920 and ≈2850 cm^−1^) were almost eliminated after repeated washing, thus confirming the successful removal of most surface ligands. X‐ray photoelectron spectroscopy (XPS) was employed to investigate the electronic state variations of CuSe, ZC, and GZC‐2 upon Zn and Ga co‐doping. As exhibited in Figure S7a (Supporting Information), the Cu 2p peaks of ZC are moved by 0.41 eV to the lower binding energy direction, compared to those of pristine CuSe. These results indicate an increased electron density around surface Cu atoms after Zn incorporation.^[^ ^24^ ^]^ The movements of Cu 2p peaks to the lower binding energy direction are more obvious for GZC‐2 (Figure S7a, Supporting Information), with the total binding energy change of 0.88 eV, compared to those of CuSe (952 and 932.12 eV corresponding to Cu 2p_1/2_ and Cu 2p_3/2_, respectively). These results indicate a further modulation of the Cu electronic environment upon Ga doping. In addition, the substitution of Cu^+^ with Zn^2+^ and Ga^3+^ introduces excess positive charges that may be compensated by the formation of Cu vacancies (V_Cu_) or other intrinsic defects, consistent with a charge‐compensation mechanism.^[^ ^25^ ^]^ Moreover, the Se 3d spectra (Figure S7b, Supporting Information) for both ZC and GZC‐2 also exhibit the movement to a lower binding energy, compared to that of CuSe. These results further imply the generation of V_Cu_ or other intrinsic defects and altered local electronic environments.^[^ ^26^ ^]^ These intrinsic defects (e.g., V_Cu_) can facilitate charge separation/transport by serving as temporary charge traps, and may also act as active centers for photocatalytic reactions.^[^ ^27^ ^]^ The Ga 2p spectrum of GZC‐2 (Figure S7c, Supporting Information) exhibits two distinct peaks at 1117.06 eV corresponding to Ga 2p_3/2_ peaks. These results reveal the successful incorporation of Ga^3+^ into the lattice. Meanwhile, the Zn 2p peaks of GZC‐2 exhibit a movement of 0.24 eV to a lower binding energy direction, compared to those of ZC (Figure S7d, Supporting Information). These results indicate that the presence of Ga^3+^ regulates the local electronic environment around Zn atoms through charge redistribution.

To further probe the valence state change of Cu, Cu K‐edge X‐ray absorption near‐edge structure (XANES) spectra were collected for CuSe, GZC‐2, and Cu foil. As exhibited in Figure 1c, the absorption edge of GZC‐2 is moved to a higher photon energy, compared to those of CuSe or Cu foil, suggesting an increased average oxidation state of Cu in the bulk after Ga and Zn incorporation. Notably, these results appear to contradict the Cu 2p spectra results (Figure S7a, Supporting Information), which exhibit shifts in Cu 2p peaks to lower binding energy upon co‐doping, and imply the reduced Cu valence state at the surface. This discrepancy highlights the spatial heterogeneity of Cu oxidation states, with surface reduction and bulk oxidation of Cu elements coexisting in GZC‐2. This is attributed to Ga^3+^‐induced surface electron enrichment and compensatory bulk oxidation. This observed discrepancy between XANES and XPS results arises from their different probing depths: XANES results reflect the bulk electronic structure utilizing high‐energy X‐rays; whereas the XPS technique is surface‐sensitive.^[^ ^28^, ^29^ ^]^ Fourier‐transformed extended X‐ray absorption fine structure (FT‐EXAFS) spectra of Cu foil, CuSe, and GZC‐2 (Figure S8, Supporting Information) reveal a similar Cu‐Se coordination (≈1.9 Å) in both CuSe and GZC‐2, indicating the preservation of CuSe host matrix after Zn and Ga incorporation. Notably, the Cu‐Se bond length in GZC‐2 is slightly shorter than in CuSe, revealing stronger Cu‐Se interactions and lattice contraction.^[^ ^30^ ^]^ This is further supported by the Morlet wavelet‐transformed (WT) spectra (Figure 1d), which confirm that Cu remains primarily coordinated to Se after doping. As exhibited in Figure 1d, the main scattering peak of GZC‐2 shifts to a lower radial distance and exhibits a reduced wave vector. This is consistent with shortened Cu‐Se bonds and a possible reduction in coordination number, owing to Zn/Ga doping and Se vacancy (V_Se_) formation.^[^ ^31^ ^]^ Ga K‐edge XANES spectra (Figure 1e) provide additional evidence. The absorption edge of GZC‐2 lies between those of Ga foil and Ga_2_Se_3_, indicating that Ga^3+^ partially substitutes for Cu and adopts a valence state slightly lower than +3. This substitution regulates the local electronic structure and defect chemistry, potentially forming partial covalent interactions with the Cu‐Se framework to strengthen bonding and enhance electron transport efficiency.^[^ ^32^ ^]^

Furthermore, the Cu L‐edge XANES spectra (Figure S9, Supporting Information) exhibit an apparent intensity enhancement of the peak at 931.5 eV for GZC‐2 compared to that of ZC or CuSe. The results indicate apparently higher proportion of Cu^2+^ states in GZC‐2. These are supported by a range of references summarized in Table S2 (Supporting Information), which clearly indicate that the peak at ≈931.5 eV arises from the existence of Cu^2+^ states in Cu L_3_‐edge XANES results.^[^ ^33^, ^34^ ^]^ This implies that Ga plays a dominant role in increasing Cu oxidation state and may facilitate defect formation, which is crucial for photocatalytic activity enhancement.^[^ ^32^, ^35^ ^]^ To gain deeper insights into the defect structures, electron paramagnetic resonance (EPR) spectroscopy was applied to explore the V_Se_ and Cu^2+^ ions in ZC and GZC‐2, respectively. The EPR spectra in Figure 1f exhibit the characteristic signals at g≈2.05 and g≈2.00, which are attributed to the unpaired electrons of Cu^2+^ (g≈2.05) and V_Se_ (g≈2.00), respectively.^[^ ^14^, ^36^ ^]^ Notably, the EPR signal intensities for both Cu^2+^ and V_Se_‐related centers are significantly increased for GZC‐2 compared to those for ZC, indicating that Ga incorporation induces a higher density of these defect states. These results are consistent with the electronic structure changes observed in the results on XPS, XANES, and EXAFS spectra, which all offer strong evidence that Zn and Ga doping promote defect engineering in GZC‐2.

Through analyzing the aforementioned XPS results (Figure S7a–d, Supporting Information), synchrotron‐based XANES results (Figure 1c,e; Figure S9, Supporting Information), and EPR results (Figure 1f) altogether, we can clarify the surface‐to‐bulk redox gradient in the GZC‐2 QDs as follows: i) The ion‐exchange process results in the replacement of Cu^+^ by Ga^3+^/Zn^2+^, particularly on the surface of GZC‐2 QDs, leading to the generation of many V_Cu_ (particularly on/near the surface of GZC‐2 QDs). The generation of these V_Cu_ is also corroborated by the obviously‐reduced Cu 2p peak intensities in the order of CuSe > ZC > GZC‐2 (Figure S7a, Supporting Information). The reduced Cu 2p signals arise from both the replacement by Ga^3+^/Zn^2+^ doptans and the formation of V_Cu_. ii) These V_Cu_ charged with negative potentials (usually with a valence status of ‐2) are generated to balance the excessive positive potentials from the doped Ga^3+^/Zn^2+^ ions. iii) Many of the Se^2−^ ions on/near the surface of GZC‐2 QDs are also oxidized to provide the electrons to balance the excessive positive potentials from the doped Ga^3+^/Zn^2+^ ions. iv) After oxidation, some Se^0^ atoms might remain on the surface of GZC‐2 QDs (Figure S7b, Supporting Information). But many Se^0^ atoms are leached out of the GZC‐2 QDs to form V_Se_. These are supported by the obviously reduced peak areas for the Se elements of GZC‐2 QDs, compared to those of CuSe (Figure S7b, Supporting Information). Besides, these are corroborated by the increased V_Se_ signals of GZC‐2, compared to those of ZC (Figure 1f). v) All the elements on/near the surface of GZC‐2 QDs (Cu, Se, Ga, and Zn elements) are partially reduced, owing to the electron‐rich local environment on/near the surface of GZC‐2 QDs. These can be strongly supported by the movements of Cu 2p, Se 3d, and Zn 2p peaks to the lower binding energy direction for GZC‐2 QDs, compared to those for CuSe or ZC QDs (Figure S7a,b; Figure S7d, Supporting Information). Also, the partial reduction of Ga^3+^ ions is corroborated by the Ga K‐edge XANES spectra results for GZC‐2 and Ga_2_Se_3_ with Ga^3+^ ions (Figure 1e). vi) To compensate for the electron accumulation on/near the surface of GZC‐2 QDs, the core/inner part of GZC‐2 QDs is oxidized and loses the electrons. This electron deficiency results in the formation of more Cu^2+^ ions at/near the core region of GZC‐2 QDs. These are revealed by the EPR results of GZC‐2 and ZC (Figure 1f), as well as the Cu K‐edge XANES results of GZC‐2 and CuSe (Figure 1c).

### 2.2 Charge Kinetics of Regulated Ga‐Zn‐Cu‐Se Quantum Dots

The band gap and band edge positions of GZC‐2 were further analyzed to investigate the roles of V_Cu_ and V_Se_ on accelerating the charge carrier kinetics. The UV‐vis absorption spectra of ZC and GZC‐2 (Figure S10a, Supporting Information) exhibit a significant enhancement of light absorption in the 200‐500 nm range, compared to that of CuSe. This enhancement can be attributed to the introduction of new electronic states, which promote stronger interactions with light in both the UV and visible regions.^[^ ^37^ ^]^ Notably, GZC‐2 exhibits slightly stronger absorption than ZC in this range (Figure S10a, Supporting Information), indicating that Ga doping contributes further to the optical properties of GZC‐2. The band edge alignment is further investigated by a range of routes. According to the UV‐vis spectra in Figure S10a (Supporting Information), the bandgaps (E_g_) were obtained from the corresponding Tauc plots (Figure S10b, Supporting Information), with values of 2.02, 2.06, and 2.10 eV for CuSe, ZC, and GZC‐2, respectively. Furthermore, the XPS‐VB (valence band) spectra of ZC and GZC‐2 (Figure S11, Supporting Information) were collected to determine the relative potential differences between the VB edge position (E_VB_) and Fermi level (E_F_), with the (E_F_‐E_VB_) values of 1.03 and 1.60 eV for ZC and GZC‐2, respectively. The Mott‐Schottky (M‐S) plots in Figure S12 (Supporting Information) exhibit the flat band potentials of ‐1.11 and ‐1.05 V versus Ag/AgCl electrode for ZC and GZC‐2, respectively. Thus, the Fermi levels of ZC and GZC‐2 are estimated to be at ‐1.11 and −1.05 V versus Ag/AgCl electrode, corresponding to ‐0.5 and ‐0.44 V versus the reversible hydrogen electrode (RHE) at pH 7. Thus, the positions of VB and conduction band (CB) for ZC and GZC‐2 are acquired and summarized in Table S3 (Supporting Information). In addition to band edge shifts, the XPS‐VB spectrum of GZC‐2 (Figure S11, Supporting Information) exhibits a shoulder peak at ≈7 eV, which is attributed to mid‐gap states arising from Ga‐ and Zn‐induced defect levels.^[^ ^38^ ^]^ These intermediate states may serve as temporary electron/hole reservoirs, enabling delayed charge carrier release for photocatalytic reaction.

To further investigate charge separation and transport mechanisms, a combination of in situ and ex situ characterization techniques was employed. Transient‐state photoluminescence (PL) spectroscopy was conducted under HMF‐soaked conditions to simulate the reaction environment. As exhibited in Figure S13a,b and Table S4 (Supporting Information), ZC exhibits reduced PL intensity and longer charge lifetimes (τ_1_ = 57.9 ns, τ_2_ = 82.6 ns, τ_a_ = 75.1 ns) compared to CuSe (τ_1_ = 56.7 ns, τ_2_ = 69.3 ns, τ_a_ = 63.6 ns), indicating enhanced charge separation owing to fewer shallow traps from Zn doping.^[^ ^39^ ^]^ This enhancement is attributed to the partial replacement of Cu by Zn, which regulates the electronic structure and defect formation. Upon Ga incorporation, GZC‐2 exhibits even longer lifetimes (τ_1_ = 65.3 ns, τ_2_ = 114.8 ns, τ_a_ = 86.9 ns), along with an increased proportion of fast decay (A_1_ = 56.4%) and decreased slow decay (A_2_ = 43.6%). These results imply Ga‐induced modulation of V_Cu_ and V_Se_ distributions and more efficient charge trapping and release kinetics.^[^ ^25^ ^]^ Ga probably promotes the formation of V_Cu_ and V_Se_, which serve as hole and electron traps, respectively, to prolong charge lifetimes and suppress recombination. After HMF adsorption, the PL intensity of GZC‐2 further decreases, with carrier lifetimes markedly extended (τ_1_ = 94.5 ns, τ_2_ = 291.7 ns, τ_a_ = 117.0 ns; Figure S13b and Table S4, Supporting Information). As exhibited in Table S4 (Supporting Information), for HMF‐GZC‐2, its fast decay component (A_1_ = 88.6%) becomes dominant, while its slow component (A_2_ = 11.4%) is apparently reduced. These results suggest that HMF molecules strongly interact with surface active sites or vacancy‐induced intermediates on GZC‐2 (and similarly on ZC), facilitating efficient hole capture.^[^ ^40^, ^41^ ^]^ These interactions prolong the overall carrier lifetimes, as observed in both HMF‐GZC‐2 and HMF‐ZC, thereby contributing to the enhanced photocatalytic activity.

To explore surface/bulk carrier kinetics, transient‐state surface photovoltage (TPV) measurements were performed and fitted with a third‐order exponential model. As exhibited in Figure S14 and Table S5 (Supporting Information), ZC exhibits a limited average lifetime (τ_a_ = 24.24 µs), attributed to significant surface traps. In contrast, GZC‐2 exhibits extended τ_1_, τ_2_, and τ_3_ components, with a longer average lifetime of 26.15 µs, reflecting improved bulk‐to‐surface charge migration enabled by V_Cu_/V_Se_ formation and Ga‐suppressed deep/surface trap states.^[^ ^42^ ^]^ Moreover, HMF adsorption reduces the average carrier lifetime in GZC‐2 (τ_a_ = 5.28 µs), primarily owing to the decrease of the long‐lived component (τ_3_ = 11.05 µs, A_3_ = 44.58%). This reveals that HMF adsorption accelerates charge extraction and transfer. The increased proportion of short‐lived carriers (A_1_ + A_2_ = 55.43%) in HMF‐GZC‐2 confirms that HMF molecules act as electron donors, facilitating hole transfer via direct interaction with surface vacancies.^[^ ^43^ ^]^ Furthermore, TPV results indicate that photo‐generated electrons migrate more efficiently from the bulk to the surface than holes, further supporting the presence of spatially directed charge kinetics in GZC‐2. Considering the electron‐accepting nature of Zn^2+^ and Ga^3+^, it is reasonable to infer that these surface‐accumulated electrons preferentially localize near Ga/Zn regions.^[^ ^44^ ^]^ This inference aligns well with the proposed charge separation model in Figure 4f, where electrons accumulate at Ga/Zn sites while holes migrate toward V_Cu_.

**Figure 2:**
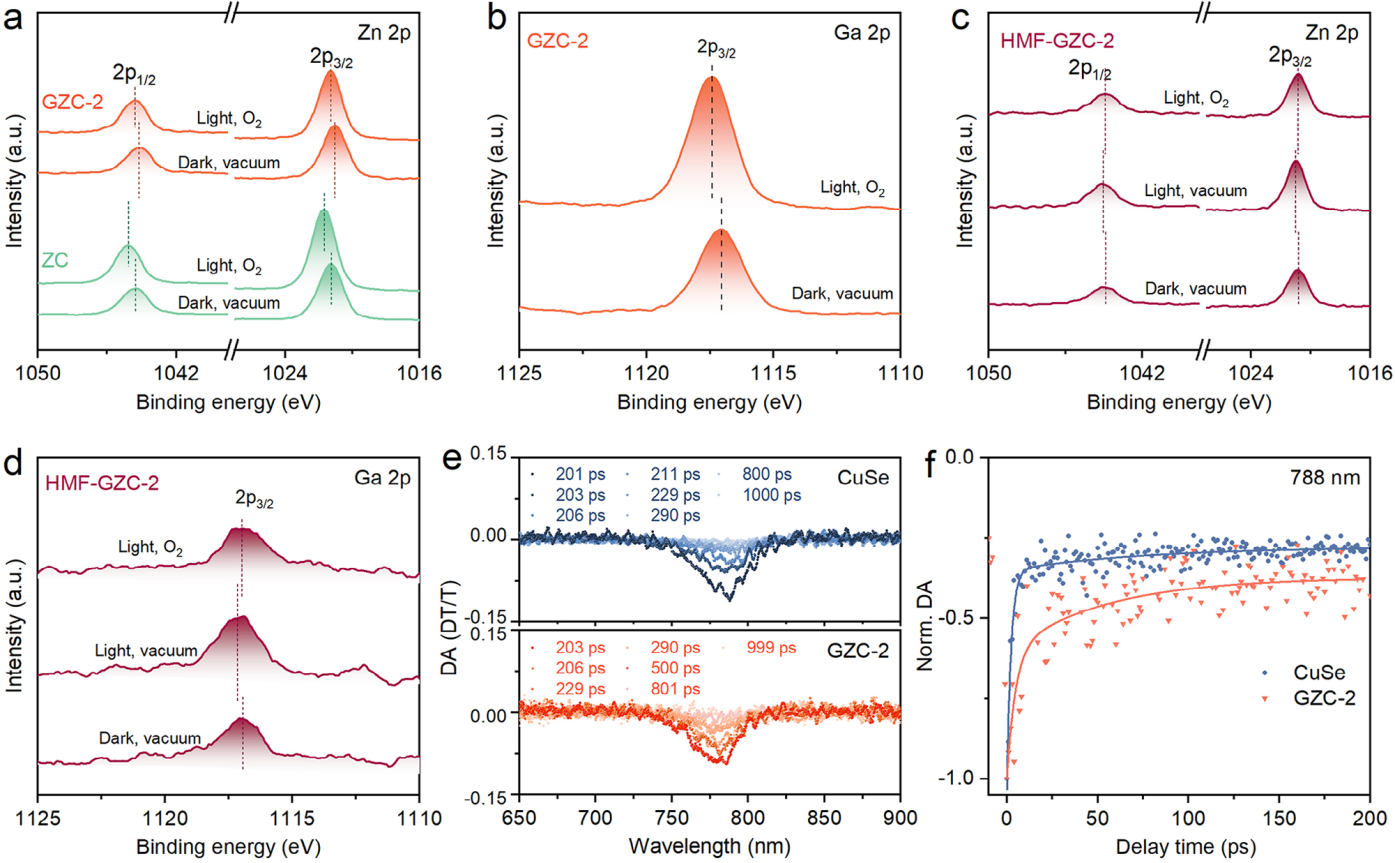
a) In situ high‐resolution XPS spectra of Zn 2p for ZC and GZC‐2 with light irradiation and in O_2_ atmosphere, compared to those in dark and vacuum conditions. b) In situ high‐Resolution XPS spectra of Ga 2p for GZC‐2 with light irradiation and in O_2_ atmosphere, compared to those in dark and vacuum conditions. In situ high‐Resolution XPS spectra of c) Zn 2p and d) Ga 2p for HMF‐GZC‐2 in the following three conditions: i) in dark and vacuum condition; ii) with light irradiation and in vacuum condition; iii) with light irradiation and in O_2_ atmosphere. e) TAS spectra for CuSe and GZC‐2, and f) their corresponding normalized decay kinetics and fitting lines taken through the GSB peaks at ≈788 nm.

The in situ XPS spectra of ZC, GZC‐2, and HMF‐GZC‐2 were collected to investigate the surface electronic environment under photocatalytic conditions. As exhibited in **Figures** **2**a,b and S15a,b (Supporting Information), the Zn, Cu, and Se peaks of ZC, as well as the Ga, Zn, Cu, and Se peaks of GZC‐2, are all moved to the higher binding energy direction under light irradiation and in O_2_ atmosphere. These results clearly reveal the presence of more holes than electrons on the whole surface of ZC and GZC‐2 in the above conditions. The reasons are explained in detail as follows: i) Under light irradiation, the photo‐excited holes tend to migrate more efficiently from the core region to the surface/near‐surface region of ZC and GZC‐2 QDs. This phenomenon occurs owing to the electron‐deficient environment at the core region and the electron‐rich environment at the surface/near‐surface region of ZC and GZC‐2 QDs. ii) In O_2_ atmosphere, the O_2_ molecules adsorbed on the surface of ZC and GZC‐2 QDs can easily attract and scavenge the photo‐excited electrons existing on the surface, mainly owing to their strong electron negativity and electron‐accepting ability. These result in the efficient consumption of photo‐excited electrons on the surface, thus contributing to the existence of more holes than electrons remaining on the surface. A careful observation of the above in situ XPS results (Figure 2a,b; Figure S15a,b, Supporting Information) reveals the more important details of these results: i) for ZC QDs, with light irradiation and in O_2_ atmosphere, the Zn 2p, Cu 2p and Se 3d peaks exhibit the movements of 0.42, 0.33 and 0.25 eV, respectively, to the higher binding energy direction (Figure 2a; Figure S15a,b, Supporting Information). ii) for GZC‐2 QDs, with light irradiation and in O_2_ atmosphere, the Zn 2p, Cu 2p, Ga 2p, and Se 3d peaks present the movements of 0.24, 0.45, 0.39 and 0.41 eV, respectively, to the higher binding energy direction (Figure 2a,b; Figure S15a,b, Supporting Information). The above detailed information is summarized in Table S6 (Supporting Information). Based on the information from Table S6 (Supporting Information), the following things can be inferred: i) Abundant photo‐excited holes accumulate around the Se/Cu elements at the surface/near‐surface of GZC‐2 QDs. ii) Fewer photo‐excited holes accumulate around Zn/Ga elements at the surface/near‐surface of GZC‐2 QDs. iii) Reason is attributed to the difference of the electron‐accepting abilities: Zn^2+^ or Ga^3+^ > Cu^+^ or Se^2−^ in the local environment at the surface/near‐surface of GZC‐2 QDs.

Nevertheless, the above in situ XPS spectra results were collected only under light irradiation and in O_2_ atmosphere. Thus, to further simulate the realistic reaction condition, the HMF soaked GZC‐2 (HMF‐GZC‐2) was tested under light irradiation and in O_2_ atmosphere to collect the in situ XPS spectra results (Figure 2c,d; Figure S16a,b, Supporting Information). A careful observation of the above results reveals the important details for HMF‐GZC‐2: i) Under light irradiation and in vacuum condition, the Zn 2p, Cu 2p, Ga 2p and Se 3d peaks exhibit the movements of 0.12, 0.22, 0.19 and 0.15 eV, respectively, to the higher binding energy direction, compared to those in dark and vacuum conditions. ii) Under light irradiation and in O_2_ atmosphere, the Zn 2p, Cu 2p, Ga 2p, and Se 3d peaks exhibit the movements of 0.11, 0.21, 0.14, and 0.13 eV, respectively, to the lower binding energy direction, compared to those under light irradiation and in vacuum conditions. The above detailed information is summarized in Table S6 (Supporting Information). Based on the information from Table S6 (Supporting Information), the following things can be inferred: i) The adsorbed HMF molecules on HMF‐GZC‐2 surface result in the apparently less movements of Zn 2p, Cu 2p, Ga 2p and Se 3d peaks to the higher binding energy direction under light irradiation and in O_2_ atmosphere, compared to the counterparts for GZC‐2 without adsorbed HMF (Table S6, Supporting Information). These results reveal that the adsorbed HMF molecules act as an effective hole scavenger to consume a major part of photo‐excited holes on the surface of GZC‐2 QDs. ii) Under light irradiation and in O_2_ atmosphere, the Zn 2p, Cu 2p, Ga 2p, and Se 3d peaks of HMF‐GZC‐2 exhibit the movements of 0.11, 0.22, 0.19, and 0.15 eV, to the lower binding energy direction, compared to those with light irradiation and in vacuum condition (Table S6, Supporting Information). These interesting results can be explained by the following reasons: i) Usually, adsorbed O_2_ molecules act as an effective scavenger to efficiently capture/consume photo‐excited electrons, resulting in more holes than electrons remaining on the surface. This well‐accepted opinion is also corroborated by the movements of Zn 2p, Cu 2p, Ga 2p, and Se 3d peaks of GZC‐2 to the higher binding energy direction under irradiation and in O_2_ atmosphere (Figure 2a,b; Figure S15a,b, Supporting Information). ii) Nevertheless, the presence of HMF molecules adsorbed on the surface of HMF‐GZC‐2 changes this trend to some extent. iii) After GZC‐2 QDs are soaked in HMF liquid, a major part of HMF‐GZC‐2 surface is covered with HMF molecules. iv) In O_2_ atmosphere, a major part of the O_2_ molecules is actually adsorbed on the HMF molecule layer rather than directly interacting with the GZC‐2 surface (Zn/Cu/Ga/Se elements). v) Thus, with light irradiation, the photo‐excited holes can be effectively captured/consumed by the adsorbed HMF molecules. And the adsorbed O_2_ molecules can apparently boost the migration of photo‐excited electrons to the surface of HMF‐GZC‐2 QDs, owing to the strong electron‐attracting ability of O_2_ molecules. vi) In comparison, a major part of the adsorbed O_2_ molecules is not that efficient to consume the electrons on the surface of HMF‐GZC‐2 QDs, because many of them do not directly interact with the GZC‐2 QDs. vii) As a result, many electrons still remain on the surface of HMF‐GZC‐2 QDs, since HMF molecules can efficiently consume the holes and existing O_2_ molecules efficiently attract but don't effectively capture the electrons. These result in the slight movements of all the Zn 2p/Cu 2p/Ga 2p/Se 3d peaks to the lower binding energy direction under light irradiation and O_2_ atmosphere, compared to those under light irradiation and in vacuum conditions for HMF‐GZC‐2 (Table S6, Supporting Information).

To further validate the role of defects, in situ EPR spectroscopy was performed for GZC‐2 and HMF‐GZC‐2 under dark or light‐irradiation conditions (Figure S17, Supporting Information). Upon HMF adsorption, intensified EPR signals for V_Se_ and Cu^2+^ were observed, revealing surface complexation and interfacial charge redistribution. Upon light irradiation, the V_Se_ signal increases in both samples, whereas the Cu^2+^ signal remains nearly unchanged. These results indicate that light‐induced formation or activation of V_Se_ plays a major role in trapping photo‐generated electrons, while Cu^2+^ contributes less to electron trapping. These results align with the XPS results, where Se 3d peaks exhibit more significant movements under light irradiation (Figure S16b, Supporting Information) compared to Cu 2p peaks (Figure S16a, Supporting Information), further confirming that V_Se_ is a key site for electron capture, enabling efficient charge separation/migration during photocatalysis.^[^ ^45^ ^]^

Transient absorption spectroscopy (TAS) was employed to investigate the influence of Ga and Zn co‐doping on charge carrier kinetics. As exhibited in Figure 2e, CuSe exhibits a strong photobleaching signal at 788 nm, indicative of substantial band‐edge electron accumulation. In contrast, GZC‐2 exhibits a weaker and blue‐shifted signal from 788 nm (in CuSe) to 778 nm, suggesting a reduction in band‐edge filling and a wider bandgap, consistent with the Tauc plot results (Figure S10b, Supporting Information). This spectral movement and intensity decrease indicate that the introduction of Ga and Zn induces V_Se_ and V_Cu_ defects, which act as charge acceptors, suppressing recombination and facilitating charge extraction.^[^ ^45^, ^46^ ^]^ Biexponential fitting of the TAS decay curves (Figure 2f; Figure S18 and Table S7, Supporting Information) reveals significant differences in carrier lifetimes. For CuSe, the fast decay component (τ_1_) is 2.32 ps at 788 nm and 1.90 ps at 778 nm, reflecting rapid charge recombination. In GZC‐2, τ_1_ increases to 4.67 ps and 3.65 ps, indicating delayed initial recombination owing to improved charge separation. The slow decay component (τ_2_) is also extended from 10.36 ps in CuSe to 87.87 ps in GZC‐2 at 778 nm, reflecting enhanced charge stability. Furthermore, the amplitude ratio shifts, with a decrease in the fast component (A_1_) and an increase in the slow component (A_2_) for GZC‐2, compared to those for CuSe.^[^ ^47^ ^]^ These results support a transition from rapid charge recombination to more sustained charge separation. The presence of V_Se_ and V_Cu_ induced by Ga‐Zn doping enhances charge separation, reduces charge recombination, and facilitates efficient charge migration, ultimately improving optoelectronic performance.

Photoelectrochemical (PEC) measurements also support the above results. As exhibited in Figure S19a (Supporting Information), GZC‐2 exhibits the highest photocurrent response among all catalysts, highlighting superior charge separation/transfer efficiency arising from V_Se_ and V_Cu_ formation. Electrochemical impedance spectroscopy (EIS) measurements (Figure S19b, Supporting Information) further confirm this enhancement: the Nyquist plots exhibit a decreasing impedance trend: CuSe > GZC‐3 > GZC‐1 > GZC‐2. GZC‐2 exhibits the smallest semicircle radius, indicating improved interfacial conductivity and lower charge transfer resistance. Overall, Ga/Zn co‐doping and the introduction of V_Cu_ and V_Se_ significantly regulate the electronic structure, enhance carrier mobility, and reduce recombination losses, collectively contributing to improved optoelectronic and photocatalytic performance.

### 2.3 Photocatalytic Performances of Regulated Ga‐Zn‐Cu‐Se Quantum Dots

**Figure 3:**
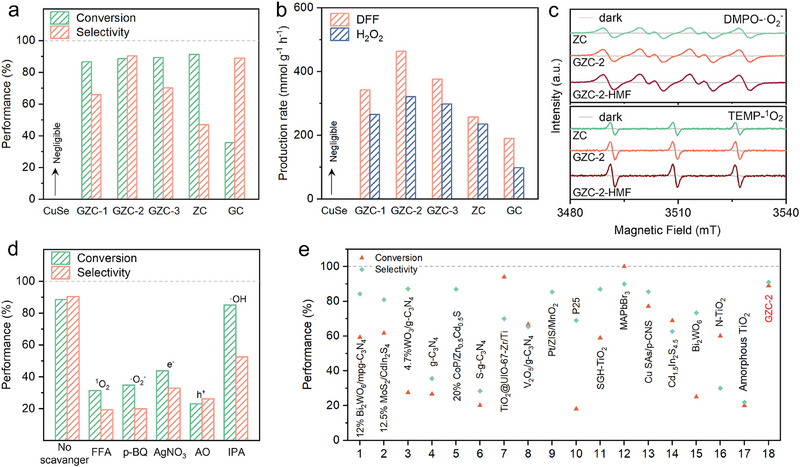
a) A comparison of HMF‐to‐DFF conversion and DFF selectivity for photocatalytic reaction utilizing various catalysts. b) The photocatalytic evolution rates of DFF and H_2_O_2_ utilizing various catalysts. c) In situ EPR spectra of DMPO‐·O_2_^−^ and TEMP‐^1^O_2_ adducts detected over various catalysts. d) Photocatalytic performances of HMF‐to‐DFF conversion and DFF selectivity over GZC‐2 after the addition of various scavengers. The exact concentrations of all the quenching agents are as follows: 0.15 mM furfuryl alcohol (FFA); 1 mM p‐benzoquinone (p‐BQ); 2 mM silver nitrate (AgNO_3_); 0.2 mM ammonium oxalate (AO); 1 vol% isopropanol (IPA). e) A comparison of photocatalytic selective HMF‐to‐DFF conversion and DFF selectivity over the reported catalysts and the optimized GZC‐2 in our report.^[^ ^48^, ^49^, ^50^, ^51^, ^52^, ^53^, ^54^, ^55^, ^56^, ^57^, ^58^, ^59^, ^60^, ^61^, ^62^ ^]^

The photocatalytic activities of various catalysts for the selective oxidation of HMF to DFF were systematically evaluated using HMF conversion and DFF selectivity as metrics. As exhibited in **Figure** **3**a, pristine CuSe exhibits negligible activity for both HMF conversion and DFF production, likely owing to its limited light absorption and ineffective charge separation arising from an unfavorable band structure. In contrast, ZC QDs achieve a high HMF conversion rate of 91%, but with a low DFF selectivity of only 47%. These results reveal that while ZC facilitates oxidation, it suffers from side reactions (e.g., over‐oxidation and byproduct formation) that compromise selectivity. Notably, as exhibited in Figure 3a, Ga‐doped CuSe (GC) exhibits a moderate performance, with only 36% HMF conversion but relatively high DFF selectivity (89%). These results reveal that doping Ga alone can apparently improve selectivity but fail to achieve a high activity for HMF conversion. All Ga/Zn co‐doped GZC quantum dots (GZC‐1, GZC‐2, and GZC‐3) retain high HMF conversion efficiency while exhibiting markedly enhanced DFF selectivity. This improvement is attributed to the introduction of V_Se_ and V_Cu_, which regulate charge carrier kinetics and increase the density of active sites. Ga doping plays a critical role in modifying the electronic structure, promoting efficient charge separation, and stabilizing intermediate species. These defects serve as selective redox centers, facilitating targeted oxidation while suppressing undesirable side pathways. Among all the GZC catalysts, GZC‐2 exhibits the best performance, achieving 89% HMF conversion and 91% DFF selectivity (Figure 3a). This reflects an optimal balance of activity and selectivity at its specific Ga/Zn doping level. The enhanced performance is associated with improved light harvesting, accelerated charge transport, and formation of reactive sites, which in turn enables controlled electron transfer and inhibit over‐oxidation. Meanwhile, the photocatalytic evolution rates of H_2_O_2_ were also assessed to provide insight into charge carrier utilization pathways (Figure 3b). CuSe exhibits negligible H_2_O_2_ evolution, while GZC‐2 presents the highest H_2_O_2_ and DFF yields among all catalysts. These results confirm that the Ga/Zn co‐doping strategy not only boosts oxidation selectivity but also enhances the two‐electron reduction of O_2_, a key step in H_2_O_2_ formation. The suppression of side reactions is further evidence of the efficient charge separation/utilization induced by defect engineering. Also, the H_2_O_2_ and DFF evolution rates of GC are exhibited in Figure 3b. Compared to ZC, GZC‐1, GZC‐2 and GZC‐3, GC presents the lowest evolution rates for H_2_O_2_ (98 mmol g^−1 ^h^−1^) and DFF (190 mmol g^−1 ^h^−1^). These results clearly reveal that doping Ga alone is much less effective compared to doping Zn alone or co‐doping of Zn and Ga. Nevertheless, GC still exhibits much higher evolution activities compared to CuSe. These results reveal that Ga doping is still an effective route to enhance the photocatalytic activity of CuSe. The long‐term stability of GZC‐2 was evaluated via repeated H_2_O_2_ production cycles (Figure S20, Supporting Information), revealing consistent activity with minimal degradation. Post‐reaction TEM analysis (Figure S21, Supporting Information) confirms that GZC‐2 maintains its morphological integrity despite slight aggregation, indicating good structural stability during photocatalysis. Collectively, these results demonstrate that Ga/Zn co‐doping enables complementary control over charge generation, transport, and surface reactivity, whereas single doping with Ga or Zn is insufficient to achieve balanced activity and selectivity. The introduction of V_Se_ and V_Cu_ effectively tailors catalytic behavior, enabling high‐performance and stable photocatalytic HMF oxidation and H_2_O_2_ generation under mild conditions.

### 2.4 Reaction Mechanism of Regulated Ga‐Zn‐Cu‐Se Quantum Dots

To elucidate the surface redox mechanism underlying the selective photocatalytic oxidation of HMF to DFF, in situ infrared (IR) spectroscopy was performed on HMF‐soaked GZC‐2 (HMF‐GZC‐2) and CuSe (HMF‐CuSe) under visible light irradiation, with O_2_ and water vapor provided. As exhibited in Figure S22a (Supporting Information), no notable IR signals can be observed for CuSe, indicating negligible HMF adsorption and surface reactivity, likely due to the absence of accessible active sites. In contrast, GZC‐2 exhibits distinct absorption bands at ≈1683, ≈1520, ≈1475, and ≈1400 cm^−1^ under visible‐light irradiation (Figure S22b, Supporting Information). We have summarized the assignments of the above four peaks in Table S9 (Supporting Information), according to the reported references. The above three peaks at ≈1683, ≈1520 and ≈1400 cm^−1^ are assigned to the C‐O stretching vibration (carbonyl group),^[^ ^63^, ^64^, ^65^, ^66^ ^]^ the conjugated C‐C stretching vibration (present in both HMF and DFF),^[^ ^63^, ^65^ ^]^ and the C‐O‐H bending modes of HMF/the C‐H wagging or scissoring modes of HMF/DFF,^[^ ^67^, ^68^ ^]^ respectively. With increasing light irradiation time (0‐40 min), the apparently increasing intensities of the peaks at ≈1683 and ≈1520 cm^−1^ are observed in Figure S22b (Supporting Information). The reasons are explained as follows: i) the increasing intensities of the peaks at ≈1683 cm^−1^ are attributed to the increased carbonyl group number in DFF molecule (2), compared to that in HMF molecule (1). ii) The increasing intensities of the peaks at ≈1520 cm^−1^ arise from the increased molecular conjugation in DFF compared to that in HMF. In comparison, the intensities of peaks at ≈1400 cm^−1^ exhibit less apparent enhancement with increasing irradiation time (Figure S22b, Supporting Information). The main reason is attributed to that both the C‐O‐H bending modes of HMF and the C‐H wagging or scissoring modes of HMF/DFF can contribute to the peaks at ≈1400 cm^−1^. Furthermore, the apparently increasing intensities of the peaks at ≈1475 cm^−1^ with increasing irradiation time are also observed in Figure S22b (Supporting Information). The reasons are explained as follows: i) The peak at ≈1475 cm^−1^ is very weak for HMF since there is only one aldehyde group (‐CHO) in HMF. ii) There are two symmetric aldehyde groups (‐CHO) in DFF, enabling the coupling of C‐H in‐plane bending for these two aldehyde groups. Thus, DFF exhibits the much stronger C‐H in‐plane bending signal in Figure S22b (Supporting Information). iii) The increased conjugation with the furan ring in DFF also contributes to the increased signal of C‐H in‐plane bending. In the (attenuated total reflectance) ATR‐FTIR spectra results, the C‐O stretching vibration peak of pure HMF appears at 1661 cm^−1^ (Figure S23, Supporting Information). In contrast, the C‐O stretching vibration peaks are moved to ≈1690 cm^−1^ for HMF‐GZC‐2 (Figure S23, Supporting Information), revealing a change in the carbonyl electronic environment. This change probably arises from the surface‐induced polarization or electrostatic interaction.^[^ ^69^ ^]^ In comparison to HMF‐CuSe, HMF‐GZC‐2 exhibits much more well‐resolved and intensified IR signals (Figure S22a,b, Supporting Information), revealing the apparently increased adsorption of HMF molecules and their conversion into DFF molecules. These arise from the synergistic effects of Ga and Zn doping into CuSe QDs.

To further investigate the reaction pathway, in situ EPR measurements were conducted on ZC, GZC‐2, and HMF‐GZC‐2 under dark and illuminated conditions (Figure 3c; Figure S25, Supporting Information). No reactive oxygen species (ROS) signals were detected in the dark, confirming the photo‐induced nature of the reaction process. Under light irradiation, GZC‐2 exhibits stronger DMPO‐·O_2_^−^ and TEMP‐^1^O_2_ signals compared to ZC, indicating that Ga incorporation enhances the generation of ROSs. This enhancement is attributed to an increased density of active sites and improved separation efficiency of photogenerated electron‐hole pairs. Furthermore, HMF‐GZC‐2 exhibits the strongest DMPO‐·O_2_^−^ and TEMP‐^1^O_2_ signals, indicating enhanced generation of both ROSs and an intensified photocatalytic interaction between HMF and GZC‐2. This enhancement is attributed to the adsorption of HMF molecules on the GZC‐2 surface, which facilitates hole capture and retains more photogenerated electrons available for O_2_ reduction, thereby promoting the formation of ·O_2_^−^ and ^1^O_2_. While ·OH radicals generation is slightly enhanced over GZC‐2 compared to that over ZC, their overall signal remains weak, indicating a minor role in the oxidation pathway and reduced chance for undesired side reactions.

To verify the function of each reactive species, trapping experiments were conducted using isopropanol (IPA), p‐benzoquinone (p‐BQ), furfuryl alcohol (FFA), ammonium oxalate (OA), and AgNO_3_ as quenchers for ·OH, ·O_2_^−^, ^1^O_2_, hole, and electron, respectively.^[^ ^70^, ^71^ ^]^ As exhibited in Figure 3d, hole quenching by OA results in the greatest suppression of HMF conversion, indicating holes as the major oxidizing species. ^1^O_2_ also plays a crucial role, exhibiting the highest contribution to DFF selectivity and secondary importance in HMF conversion. ^1^O_2_ generation was further confirmed by in situ EPR (Figure 3c), highlighting its key role in promoting DFF selectivity. Mechanistically, ^1^O_2_ originates from energy transfer between triplet oxygen and electrons accumulated at Ga^3+^/Zn^2+^ sites. ·O_2_^−^ also significantly affects both conversion and selectivity. In contrast, AgNO_3_ quenching suppresses both metrics, confirming the importance of electrons in driving the O_2_ reduction to ·O_2_^−^. IPA addition results in negligible changes, reaffirming that ·OH radicals contribute minimally to the selective oxidation pathway. Based on these quenching results, we can deduce that the importance of active species in the GZC‐2 system for photocatalytic HMF‐to‐DFF conversion can be arranged in this order: i) for HMF conversion, hole > ^1^O_2_ > ·O_2_^−^ > electron > ·OH; ii) for DFF selectivity, ^1^O_2_ > ·O_2_^−^ > hole > electron > ·OH. These insights collectively confirm that the selective HMF‐to‐DFF conversion over GZC‐2 proceeds via a ROS‐mediated pathway involving hole, ^1^O_2_, and ·O_2_^−^, with minimal involvement of ·OH. Finally, to objectively evaluate the optimized performance in our report, the photocatalytic activity and selectivity of GZC‐2 are compared with previously reported catalysts (Figure 3e). Compared with literature‐reported systems, GZC‐2 achieves the outstanding balance between HMF conversion and DFF selectivity (Figure 3e), ranked as almost the most active/selective photocatalyst for HMF‐to‐DFF conversion to date. A detailed comparison of key reaction parameters and performance metrics is provided in Table S8 (Supporting Information) for the objective and impartial comparison. Many of the reported works suffer from a trade‐off between high conversion and low selectivity owing to uncontrolled oxidation pathways. In contrast, GZC‐2 achieves both high HMF conversion and high DFF selectivity under mild conditions, outperforming most reported systems. This superior performance arises from the synergistic effects of Ga/Zn co‐doping and the formation of V_Cu_ and V_Se_, which collectively enhance charge separation and selective oxidation, while minimizing over‐oxidation.

**Figure 4:**
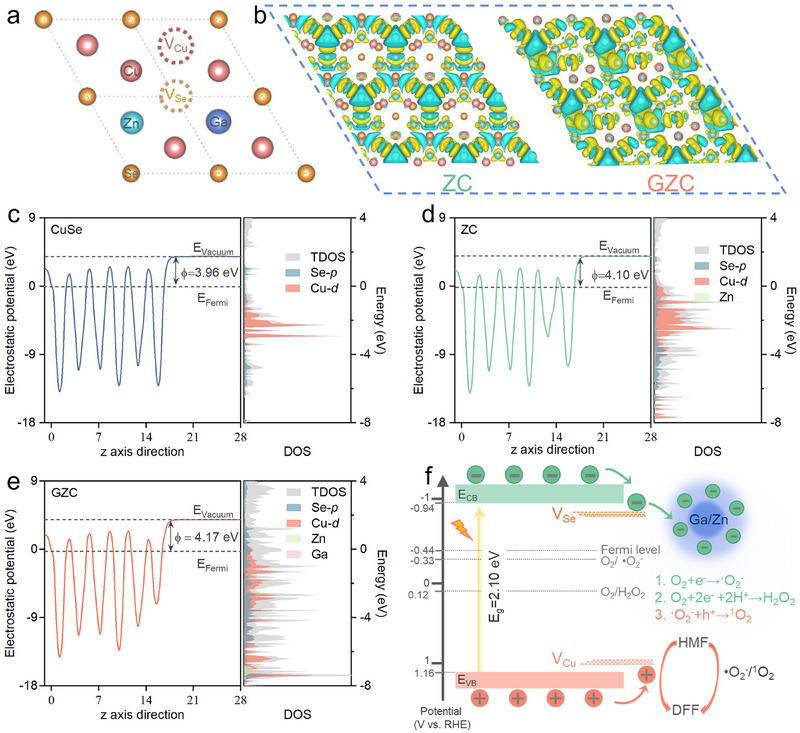
a) Schematic illustration of V_Cu_ and V_Se_, along with Zn and Ga substitutional doping on the CuSe (001) facet. b) Charge density differential maps of ZC and GZC, exhibiting electron redistribution upon doping. Plane‐averaged electrostatic potential profiles along the *z*‐axis direction and corresponding density of states for c) CuSe, d) ZC, and e) GZC. f) Raised mechanism on photocatalytic selective oxidation of HMF into DFF coupled with H_2_O_2_ evolution on GZC‐2.

To further understand the role of Zn and Ga co‐doping in regulating the atomic and electronic structure of GZC‐2 and its correlation with photocatalytic activity, density functional theory (DFT) calculations were performed. A schematic illustration of structural modifications upon Ga and Zn incorporation into the CuSe lattice is exhibited in **Figure** **4**a. The replacement of Cu^+^ by Zn^2+^/Ga^3+^ induces a charge imbalance, which is compensated by the formation of V_Cu_ and V_Se_ to maintain electrostatic neutrality. In particular, Ga^3+^, owing to its higher valence, promotes the generation of both types of vacancies to stabilize the lattice. Charge density difference plots (Figure 4b) reveal a relatively uniform charge distribution in ZC. In comparison, GZC‐2 exhibits charge localization near Se sites, indicating stronger electronic polarization and enhanced V_Se_ formation. This reveals improved charge separation and increased catalytic reactivity. The calculated work functions (WF) of CuSe, ZC, and GZC‐2 exhibit a progressive increase (CuSe < ZC < GZC), indicating enhanced electron retention and suppressed recombination.^[^ ^72^ ^]^ This increase is expected to promote charge polarization, leading to hole‐enriched regions in the vicinity of Ga sites.^[^ ^73^ ^]^ As exhibited in Figure 4c, CuSe exhibits the lowest WF, correlating with rapid electron loss and poor photocatalytic stability. Upon Zn doping, the WF increases (Figure 4d), indicating enhanced surface stability and reduced electron leakage. The replacement of Cu by Zn induces local lattice distortions and favors the formation of V_Cu_, which serves as a transient hole trap, thereby promoting charge separation. Further Ga doping results in the highest WF (Figure 4e), revealing a deeper Fermi level and enhanced interfacial charge transfer capacity, owing to additional vacancy formation and surface reconstruction. Density of states (DOS) and projected DOS (PDOS) analyses further corroborate these observations. As exhibited in Figure 4c, for CuSe, narrow bandgaps and strong Cu‐d/Se‐p orbital overlap near the Fermi level result in strong hole localization and rapid charge recombination. This results in poor charge separation, restricting its photocatalytic efficiency. Zn doping introduces additional states, regulates the band structure, and increases the bandgap width, accelerating charge separation by disrupting localized Cu‐d states (Figure 4d). As exhibited in Figure 4e, co‐doping with Ga and Zn further broadens the bandgap, enhances charge redistribution, and introduces mid‐gap states associated with V_Cu_ and V_Se_, thereby improving charge mobility and lowering recombination losses.^[^ ^74^, ^75^ ^]^ These results reveal that dual‐vacancy engineering via Ga/Zn co‐doping effectively regulates the electronic structure of GZC‐2, prolongs carrier lifetimes, and improves utilization of photo‐generated charge carriers.

Based on experimental characterizations and theoretical calculation results, a mechanistic model for GZC‐2 is proposed (Figure 4f). Under visible‐light irradiation, electrons are excited from the VB to the CB of GZC‐2, leaving behind photogenerated holes. The shallow energy levels introduced by V_Se_, located slightly below the CB edge, can temporarily accommodate these electrons from the CB of GZC‐2. Then, these electrons migrate to the nearby Ga/Zn sites, owing to their good electron‐extracting abilities. After that, these electrons can reduce the O_2_ molecules adsorbed on GZC‐2 to generate H_2_O_2_ as the reduction product or produce ∙O_2_^−^_,_ which will further be involved in the reactions. Meanwhile, photo‐excited holes in the VB of GZC‐2 are transferred to the shallow energy levels created by V_Cu_, slightly above the VB edge. These shallow energy levels can serve as hole‐trapping centers and mediate the transport of holes to the adsorbed HMF molecules. Then, these holes can directly oxidize the HMF into DFF as the oxidation product. Besides, these holes can also oxidize the ∙O_2_^−^ to produce ^1^O_2_, which can further oxidize the HMF into DFF. Furthermore, the generated ∙O_2_^−^ from the reduction reaction can also directly oxidize the HMF into DFF. The importance of the above oxidation species for HMF‐to‐DFF conversion is in the order of hole > ∙O_2_^−^ > ^1^O_2_, as confirmed by the quenching experiment results (Figure 3d). Overall, this dual‐vacancy strategy via Ga/Zn co‐doping enables GZC‐2 to achieve both highly selective HMF‐to‐DFF conversion and efficient H_2_O_2_ production under mild photocatalytic conditions.

## 3 Conclusion

Our research reports the preparation of a novel Ga‐Zn co‐doped Ga‐Zn‐Cu‐Se quantum dot photocatalyst (GZC‐2), through a dual‐vacancy engineering strategy via Zn/Ga co‐doping in the CuSe template. The optimized GZC‐2 exhibits excellent performance for the selective oxidation of 5‐hydroxymethylfurfural (HMF) into 2,5‐diformylfuran (DFF), with 89% HMF conversion and 91% DFF selectivity. Meanwhile, GZC‐2 also achieves an excellent H_2_O_2_ evolution activity (321 µmol g^−1^ h^−1^). The remarkable performances are attributed to Zn/Ga dopants and the induced Se vacancies (V_Se_) and Cu vacancies (V_Cu_) in GZC‐2. Theoretical calculations reveal that Zn/Ga co‐doping increases the work function, enhances charge separation, stabilizes the surface, and expands the bandgap, as supported by density of states (DOS) analysis and differential charge density mapping. Moreover, the presence of V_Se_ and V_Cu_ regulates charge separation/transfer and enhances reactive oxygen species (ROS) generation, which are validated by in situ X‐ray photoelectron spectroscopy (XPS), transient absorption spectroscopy (TAS), transient‐state photoluminescence (PL) spectroscopy, transient‐state surface photovoltage (TPV) spectroscopy, and in situ electron paramagnetic resonance (EPR) spectroscopy. The species quenching experiments clearly reveal that, among all the active species, holes play the most important role in efficient HMF‐to‐DFF conversion. Overall, the synergy between theoretical and experimental results confirms that Ga‐Zn co‐doping effectively regulates the electronic environment, stabilizes Cu oxidation states, and optimizes charge kinetics. This research exhibits a rational design strategy for advancing vacancy‐engineered photocatalysts in sustainable biomass conversion and green chemical generation.

## Conflict of Interest

The authors declare no conflict of interest.

## Supporting information



Supporting Information

## Data Availability

The data that support the findings of this study are available from the corresponding author upon reasonable request.
