# Supplementary material for: Dual Vacancy Engineering in Alloyed Ga‐Zn‐Cu‐Se Quantum Dots for Photocatalytic 5‐Hydroxymethylfurfural to 2,5‐Diformylfuran Conversion
Source: Adv Mater. 2025 Aug 27;37(45):e10164. doi: 10.1002/adma.202510164 (PMC12617044; doi:10.1002/adma.202510164)
Supplement: Supplementary file 1 — Supporting Information [file ADMA-37-e10164-s001.docx]

Supporting Information

**Dual Vacancy Engineering in Alloyed Ga-Zn-Cu-Se Quantum Dots for Photocatalytic 5-Hydroxymethylfurfural to 2,5-Diformylfuran Conversion**

Meijun Guo, Tianyu Zhao, Shuangming Chen, Li Song, Bingquan Xia, Jingrun Ran*, Shi-Zhang Qiao*

M. Guo, Dr. J. Ran, Prof. S.-Z. Qiao

School of Chemical Engineering, The University of Adelaide, Adelaide, SA 5005, Australia

E-mail: jingrun.ran@adelaide.edu.au; s.qiao@adelaide.edu.au

T. Zhao

School of Chemistry and Chemical Engineering, Harbin Institute of Technology, Harbin 150001, People’s Republic of China

Prof. S. Chen, Prof. L. Song

National Synchrotron Radiation Laboratory, CAS Centre for Excellence in Nanoscience, University of Science and Technology of China, Hefei 230029, People’s Republic of China

Dr. B. Xia

Key Laboratory for Green Chemical Process of Ministry of Education, School of Chemistry and Environmental Engineering, Wuhan Institute of Technology, Wuhan 430074, People’s Republic of China.

**1. Materials**

**(1) Chemicals**

Selenium powder (Se, 99.95%), 1-dodecanethio (≥98%), oleylamine (OLA, 70%), copper (II) acetylacetonate (Cu(acac)_2_, 97%), zinc acetate (Zn(OAc)_2_, ≥98%), octylamine (OctAm, 99%), oleic acid (OA, ≥99%), gallium(III) nitrate (Ga(NO_3_)_3_, 99.9%), 1-octadecene (technical grade, 90%), trioctylphosphine (TOP, 97%), toluene (99.5%) were purchased from Sigma-Aldrich. All chemicals were used as received without further purification. The water used in all experiments was deionized. All chemicals were used as received without further purification.

**(2) Preparation of** **CuSe templates**

First, a Se precursor was prepared as follows: Se powder (10 mM) was mixed with 5 mL of DDT and 5 mL of OLA, then degassed under N_2_ at 50 °C for 1 h. The Se powder completely dissolved via reduction by DDT, forming a brown stock solution of alkylammonium selenide, which was cooled to room temperature and stored in an N_2_-filled glovebox. For the CuSe synthesis, Cu(acac)_2_ (2 mmol) was combined with 6 mL of DDT and 19 mL of OLA in a 100 mL three-neck round-bottom flask, then degassed under N_2_ with vigorous stirring at 60 °C for 1 h. The temperature was then rapidly increased to 200 °C, where Cu(acac)_2_ fully dissolved, yielding a clear yellow-orange solution. At this point, a mixture of 2 mL of the Se precursor (2 mM Se) and 3 mL of DDT was swiftly injected into the flask, causing an immediate color change from orange to greenish-brown. The reaction proceeded at 200 °C for 3 min before being cooled using ice water. To isolate the CuSe templates, the crude solution was centrifuged at 3,000 rpm, and the supernatant was collected and further centrifuged at 8,000 rpm. The resulting precipitate was washed three times with toluene under 8,000 rpm centrifugation and finally stored in 3 mL of toluene inside a glovebox.

**(3) Preparation of ZC QDs**

First, a Zn precursor was prepared as follows: Zn(OAc)_2_ (10mM) was diluted in 5 mL of OlAm and 5 mL of OctAm in a 100 mL three-neck round-bottom flask, then degassed under N_2_ with vigorous stirring at 60 °C for 1 h. The temperature was then increased to 150 °C to dissolve all the Zn(OAc)_2_ and further kept at 60 °C. In a typical cation exchange process, 1 mL of the CuSe in toluene, 0.3 mL of Zn precursor and 2 mL of TOP was injected in previously degassed ODE (12 mL) at 150 °C under inert gas. The mixture was kept stirring at 150 °C for 10 min after the injection.

**(4) Preparation of GC QDs**

First, a Ga precursor was prepared as follows: Ga(NO_3_)_3_ (10 mM) was diluted in 10 mL of ODE and 5 mL of OA in a 100 mL three-neck round-bottom flask, then degassed under N_2_ with vigorous stirring at 60 °C for 1 h. The temperature was then increased to 150 °C to dissolve all the Ga(NO_3_)_3_ and further kept at 60 °C. In a typical cation exchange process, 1 mL of the CuSe (~0.3 mM) in toluene, 0.15 mL of Ga precursor and 2 mL of TOP was injected in previously degassed ODE (12 mL) at 150 °C under inert gas. The mixture was kept stirring at 150 °C for 10 min after the injection.

**(5) Preparation of GZC QDs**

First, a Ga precursor was prepared as follows: Ga(NO_3_)_3_ (10 mM) was diluted in 10 mL of ODE and 5 mL of OA in a 100 mL three-neck round-bottom flask, then degassed under N_2_ with vigorous stirring at 60 °C for 1 h. The temperature was then increased to 150 °C to dissolve all the Ga(NO_3_)_3_ and further kept at 60 °C. In a typical cation exchange process, 1 mL of the CuSe (~0.3 mM) in toluene, 0.15 mL of Zn precursor, 0.15 mL of Ga precursor and 2 mL of TOP was injected in previously degassed ODE (12 mL) at 150 °C under inert gas. The mixture was kept stirring at 150 °C with sampling at 5 min (GZC-1), 10 min (GZC-2) and 15 min (GZC-3) after the injection.

After synthesis, all the crude quantum dot suspension were washed and centrifuged three times with *n*-hexane and methyl acetate, respectively, to remove residual surface ligands before characterization and photocatalytic test.

**2. Methods**

**(1) Physicochemical characterizations**

The XRD patterns were acquired on a silicon substrate on a powder X-ray diffractometer (D4 ENDEAVOR, Bruker) utilizing Co Kα radiation. Transmission electron microscope (TEM) images were obtained on a FEI Tecnai G2 Spirit TEM (Thermo Fisher Scientific, USA). High-angle annular dark-field scanning transmission electron microscopy (HADDF-STEM) images, and EDS mapping images were taken on a FEI Titan Themis 80-200 (200 kV voltage) equipped with a Gatan Quantum GIF 965 Electron energy loss spectrometer for rapid compositional analysis. A K-Alpha plus XPS system (Thermo Fisher Scientific, USA) was used for XPS measurement. A light emitting diode was adopted as the light source to excite the photocatalysts in the in-situ XPS measurements. *In situ* FTIR spectra were determined using a Nicolet iS20 spectrometer equipped with an HgCdTe (MCT) detector cooled with liquid nitrogen. The synchrotron radiation based XANES measurements were conducted at the Australian Synchrotron. The UV-Vis diffuse reflectance spectra were obtained on a UV-Vis spectrophotometer (UV2600, Shimadzu, Japan). Steady-state photoluminescence (PL) spectra were obtained on a RF-5301PC spectro fluorophotometer (Shimadzu, Japan). The transient-state PL spectra were obtained on a FLS1000 spectrometer (Edinburgh Instruments, UK). The electron spin resonance (ESR) spectra were tested with ESR spectrometer (Bruker model A300).

**(2) Photocatalytic activity test**

10 mg of the as-prepared catalyst was dispersed in 20 mL of aqueous HMF solution (0.5 mM) without any sacrificial reagents. The suspension was stirred in the dark for 30 min while continuously bubbling high-purity O_2_ to ensure O_2_ saturation. After that, the reactor was sealed to maintain a dissolved oxygen atmosphere throughout the reaction. Photocatalytic reactions were carried out in a custom-designed, three-neck quartz reactor consisting of a cylindrical main body (70 mm in diameter, 45 mm in height). A 300 W xenon lamp (PLS-SXE300C, Perfectlight) equipped with a UV cut-off filter (λ > 420 nm) was vertically positioned above the reactor. The incident light intensity at the liquid surface was measured to be 125 mW cm^-2^ using a calibrated optical power meter. During irradiation, the suspension was magnetically stirred continuously to maintain uniform dispersion. All reactions were conducted at ambient temperature (~25 °C). After 2 hours of visible-light irradiation, the suspension was filtered through a 0.22 μm membrane to remove the catalyst. The resulting supernatant was collected and subjected to further analysis. Then after dilution and filtration, the reacted solution was injected into a high-performance liquid chromatograph (HPLC) equipped with a Aminex HPX-87HHPLC Column (7.8 mmx300 mm) at·265 ·nm ·wavelength for HMF and DFF quantification. The column was maintained at a temperature of 50°C and employed a binary gradient pumping method using 5 mM H_2_SO_4_. The flow rate was set at 0.6 mL/min for 90 min. The HMF conversion and the DFF selectivity are calculated according to the following equations.

$$\text{Conversion (\%)=[(}\text{C}_{\text{0}}\text{-}\text{C}_{\text{HMF}}\text{)/}\text{C}_{\text{0}}\text{]×100}$$

$$\text{Selectivity (\%)=[}\text{C}_{\text{DFF}}\text{/(}\text{C}_{\text{0}}\text{-}\text{C}_{\text{HMF}}\text{)]×100}$$

where C_0_ is the initial concentration of HMF and C_HMF_ and C_DFF_ are the concentrations of the substrates HMF and DFF after reaction, respectively.

The H_2_O_2_ amount was determined by redox titration with KMnO_4_ (0.2 mM) with the addition of 5 mL of a 1 M H_2_SO_4_ solution. Add 5 mL of 1 M H_2_SO_4_ to the flask containing the H_2_O_2_ sample. The acid provides the acidic medium necessary for the redox reaction. Then, fill the burette with 0.2 mM KMnO_4_ solution. Gradually add KMnO_4_ solution to the acidic H_2_O_2_ solution while continuously swirling the flask until a persistent pink color is observed. This indicates the endpoint of the titration. The redox reaction between H₂O₂ and KMnO_4_ in acidic solution can be represented as:

$$\text{5}\text{H}_{\text{2}}\text{O}_{\text{2}}\text{+2KMn}\text{O}_{\text{4}}\text{+3}\text{H}_{\text{2}}\text{SO}_{\text{4}}\text{→2Mn}\text{SO}_{\text{4}}\text{+}\text{K}_{\text{2}}\text{SO}_{\text{4}}\text{+5}\text{O}_{\text{2}}\text{+8}\text{H}_{\text{2}}\text{O}$$

Then the initial and final burette readings are recorded to determine the volume of KMnO_4_ solution used in the titration. By following the above titration method and using the stoichiometry of the redox reaction, the amount of H_2_O_2_ in the sample can be accurately determined.

**(3) Photoelectrochemical Experiments**

An electrochemical analyzer (CHI760E instruments) was used to conduct electrochemical impedance spectroscopy (EIS), transient photocurrent curves and Mott-Schottky plots in 0.5 M Na_2_SO_4_ aqueous solution using a standard three-electrode system. The light source was a 300 W Xenon light with a UV-cutoff filter (λ > 400 nm). The working electrode was prepared as follows: 10 mg sample, 15 mg polyethylene glycol (PEG, molecular weight: 20000) and 1.0 mL ethanol were ground together to make a slurry. 100 μL of the slurry was coated onto a 16 mm × 12 mm F-doped tin oxide (FTO) glass-electrode using a doctor-blade. The coated electrode was dried and heated at 350 °C for 0.5 h under flowing UHP Ar flow. All electrochemical experiments were carried out in a three-electrode glass cell with a carbon-rod as the counter-electrode, and an Ag/AgCl as the reference electrode (Pine Research Instrumentation).

**(4) Theoretical computations**

All electronic structure computations were conducted using density functional theory (DFT) methods with the Perdew-Burke-Ernzerhof (PBE) generalized gradient approximation (GGA) exchange correlation functional, with a projector augmented-wave method in the Vienna Ab initio Simulation Package (VASP) code. For the plane-wave expansion, a 450 eV kinetic energy cutoff was set according to different cutoff energy tests. The convergence criteria for the geometric optimization and energy calculation was as follows: (i) self-consistent field energy tolerance of 1.0 × 10^-4^ eV, and (ii) all atoms were fully relaxed the total energy is smaller than 1.0 × 10^-3^ eV/ Å between two ionic steps. The K-points were set to 1 × 1 × 1 for the surface model. A vacuum-slab with thickness of 20 Å was applied to obviate influence of the periodic boundary. The structural optimization parameters for acquiring the work functions (Φ) are shown as follows: (0 0 1) facet of CuSe, ZC and GZC (space group P6₃/mmc): a = 8.0417 Å, b = 8.0417 Å, c = 41.2702 Å, α = β = 90º, γ = 120º, K-points setting of 1 × 1 × 1. The structural optimization parameters for acquiring the differential charge density map of CuSe, ZC and GZC (0 0 1) structure are shown as follows: (0 0 1) facet of CuSe, ZC and GZC (space group P6₃/mmc): a = 8.0417 Å, b = 8.0417 Å, c = 41.2702 Å, α = β = 90º, γ = 120º, K-points setting of 1 × 1 × 1. The structural optimization parameters for acquiring the density of states (DOS) of CuSe, ZC and GZC (0 0 1) structure are shown as follows: (0 0 1) facet of CuSe, ZC and GZC (space group P6₃/mmc): a = 8.0417 Å, b = 8.0417 Å, c = 41.2702 Å, α = β = 90º, γ = 120º, K-points setting of 3 × 3 × 3.

**
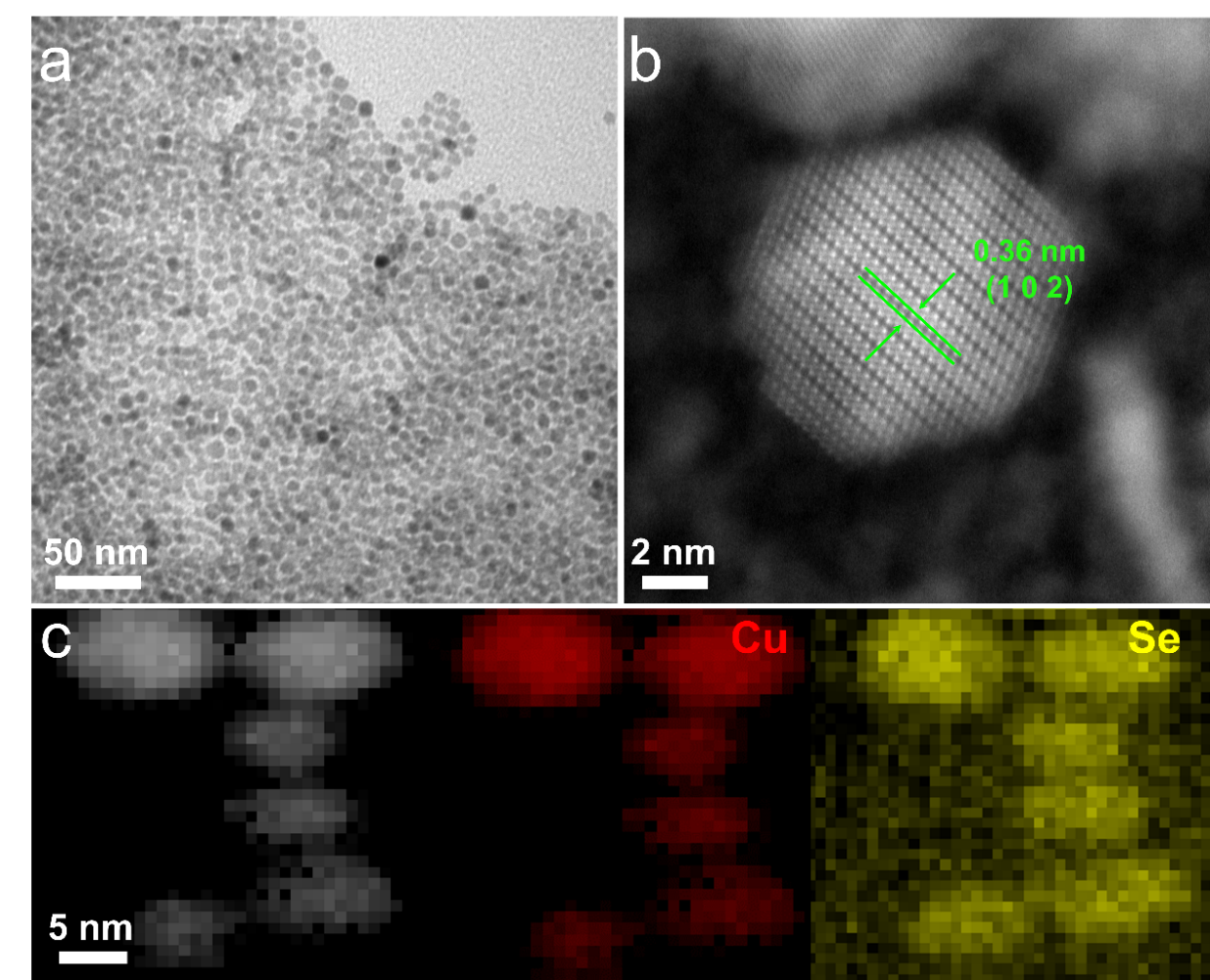
**

**Figure S1**. (a) TEM image, and (b) HAADF-STEM image of CuSe templates. (c) ADF image of the mapped area recorded simultaneously with the EELS mapping images of Cu and Se elements for CuSe template.


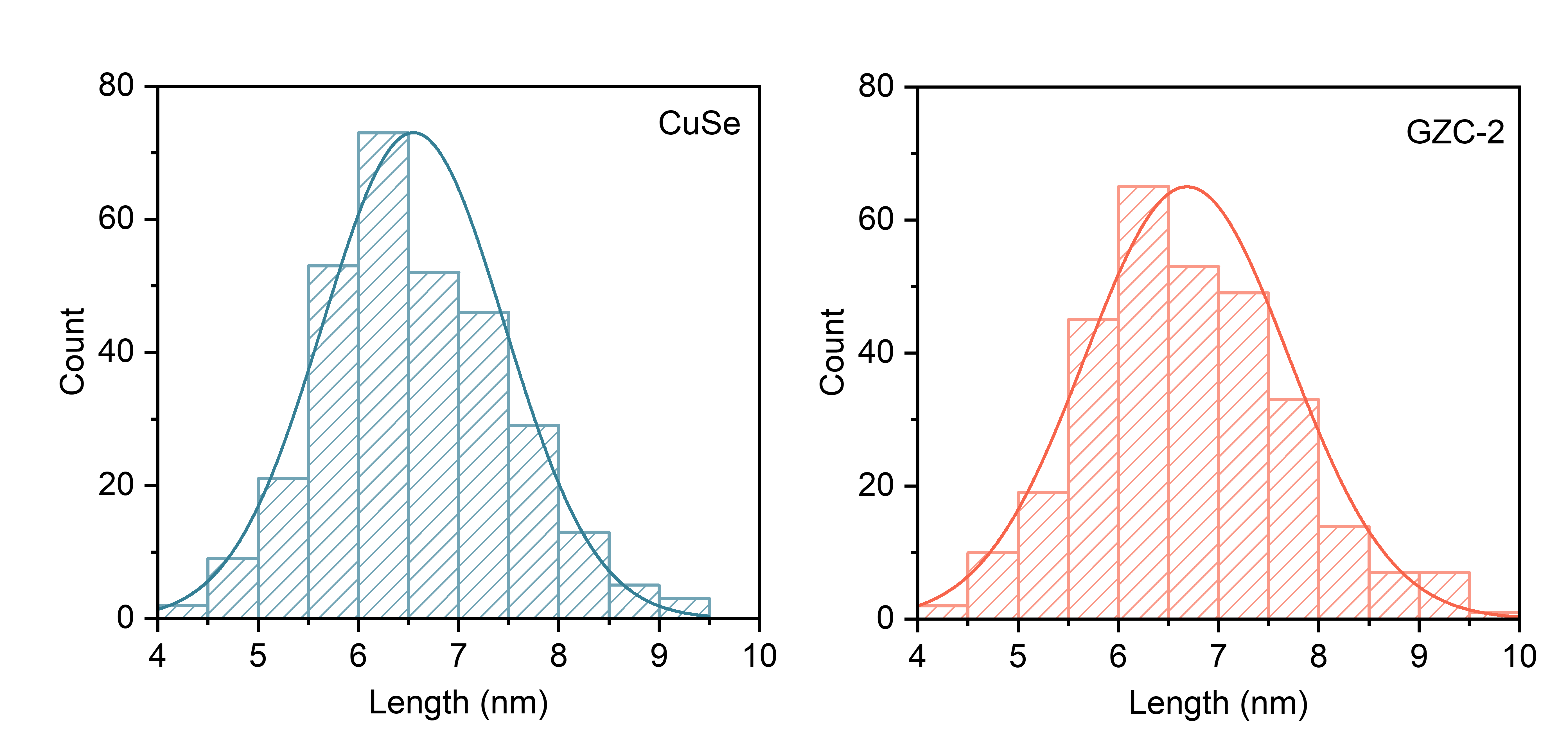


**Figure S2**. Results on particle size analysis of CuSe QDs.


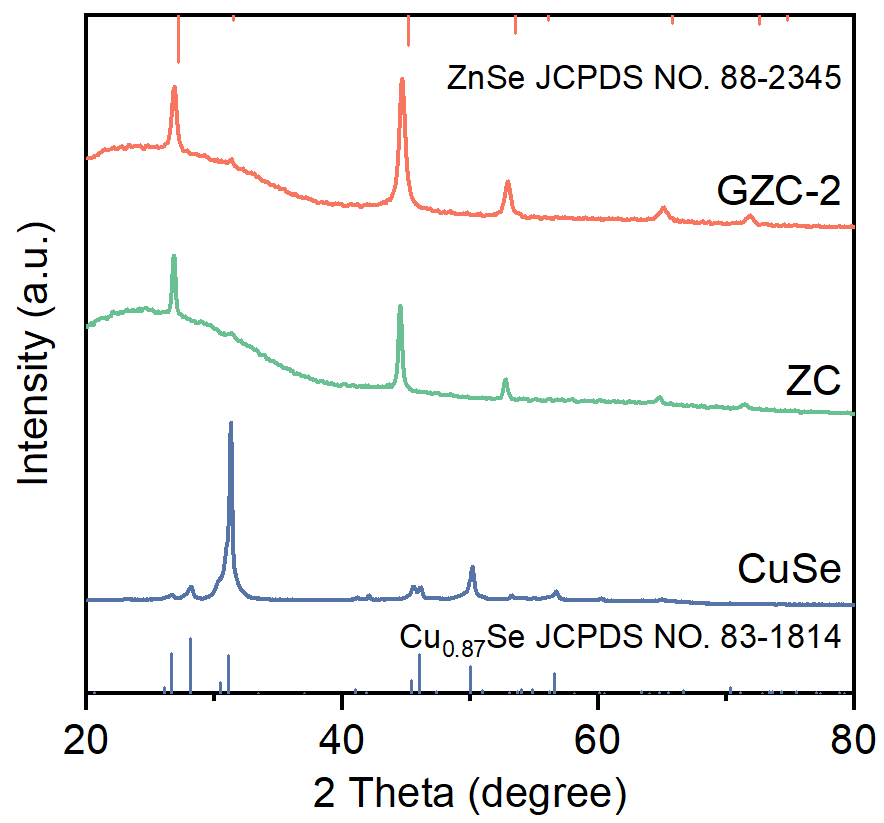


**Figure S3**. XRD patterns of GZC-2, ZC and CuSe QDs.


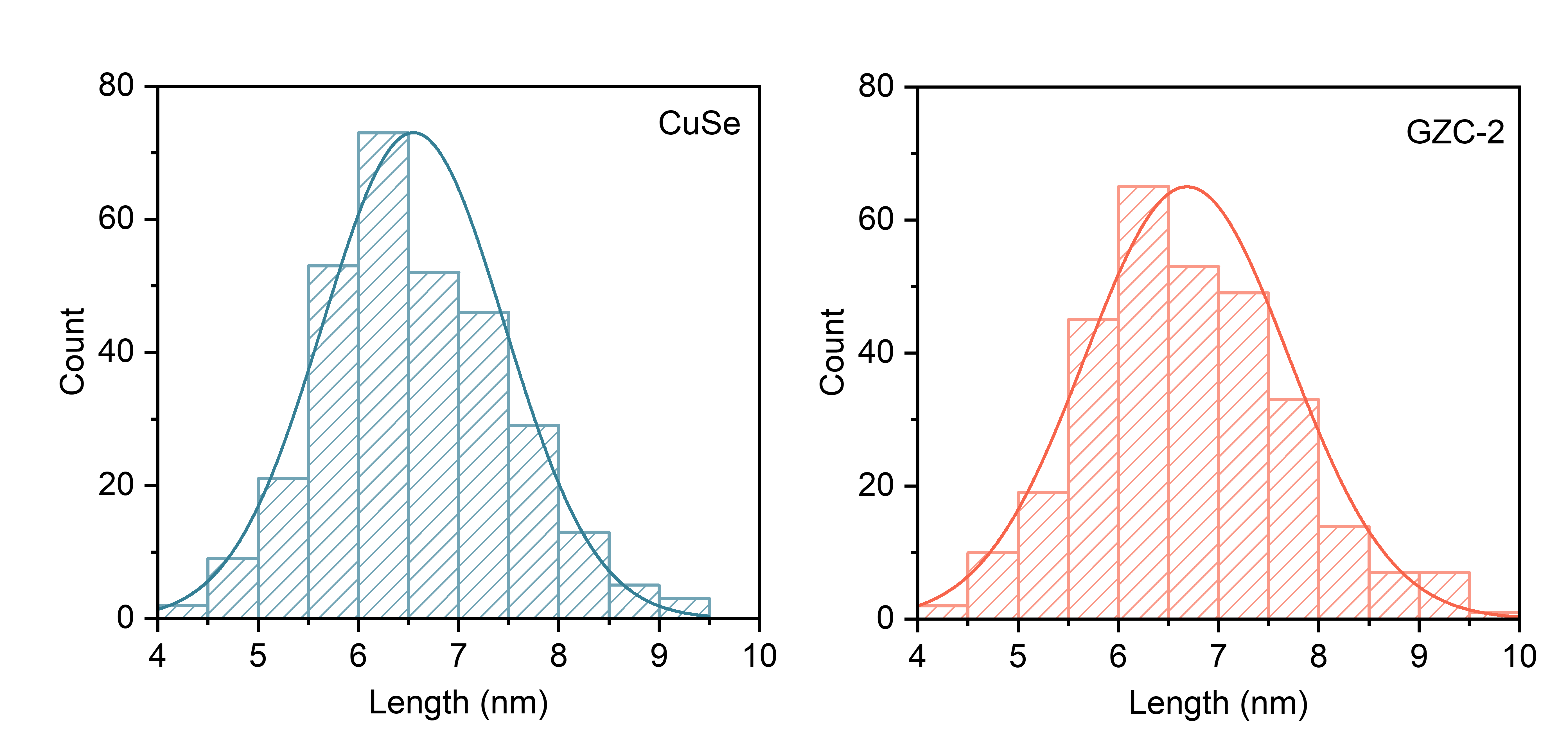


**Figure S4**. Results on particle size analysis of GZC-2.


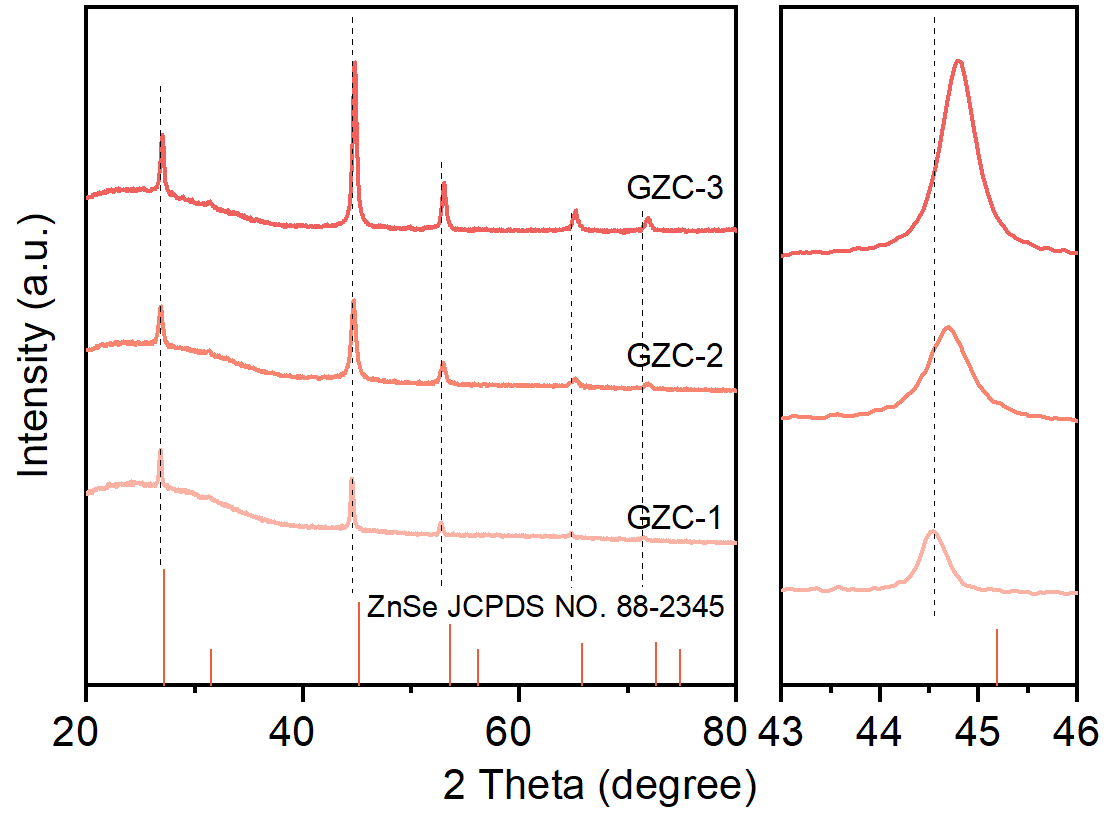


**Figure S5**. XRD patterns of as-prepared GZC-1, GZC-2 and GZC-3.


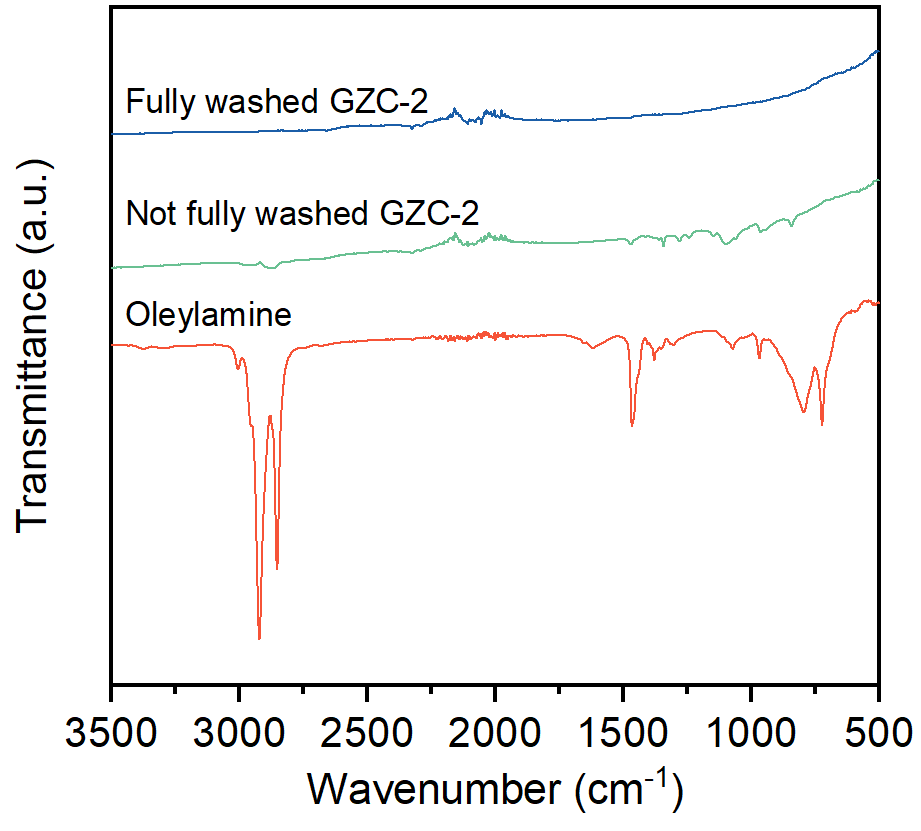


**Figure S6**. FTIR spectra of oleylamine, the fully washed GZC-2 QDs (washed three times by h-hexane and methyl acetate, respectively) and not fully washed GZC-2 QDs (washed once by h-hexane).


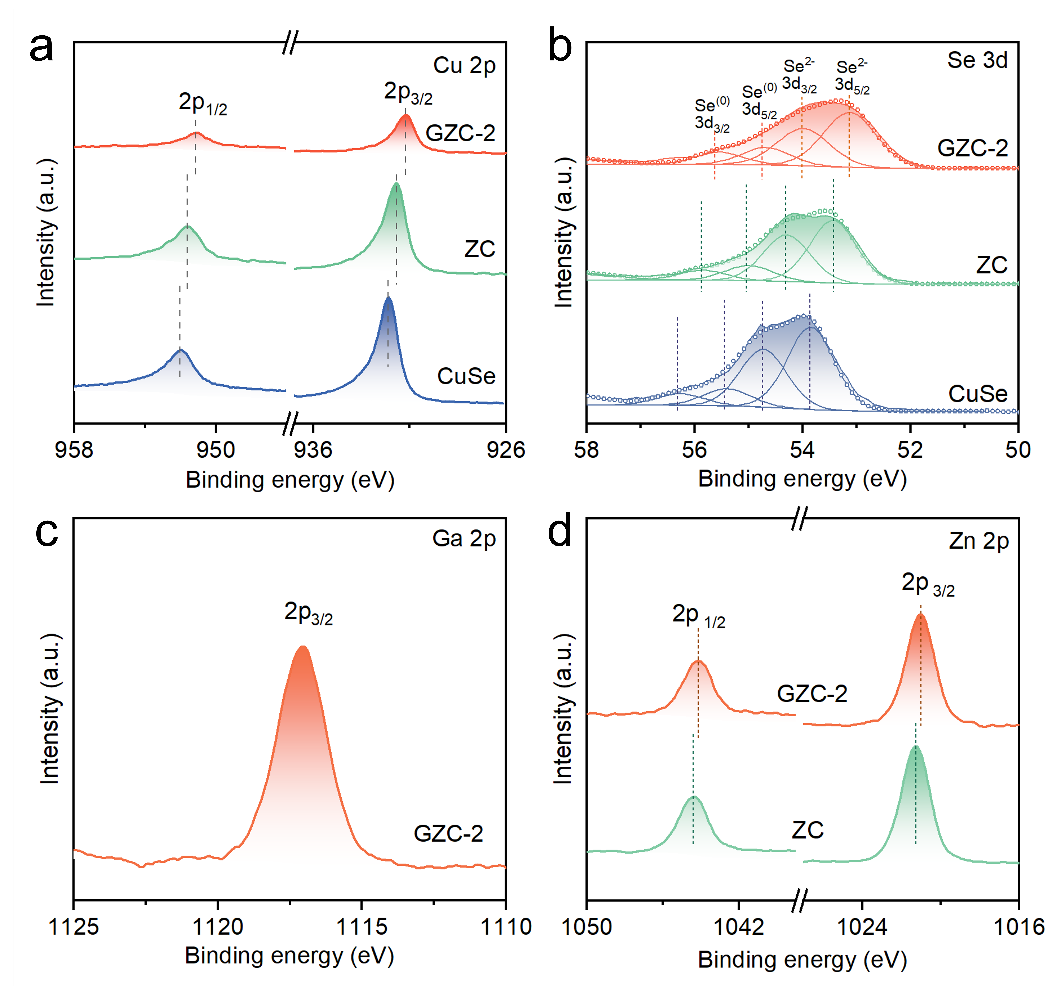


**Figure S7**. XPS spectra of (a) Cu 2p, (b) Se 3d, (c) Ga 2p and (d) Zn 2p for CuSe, ZC and GZC-2.


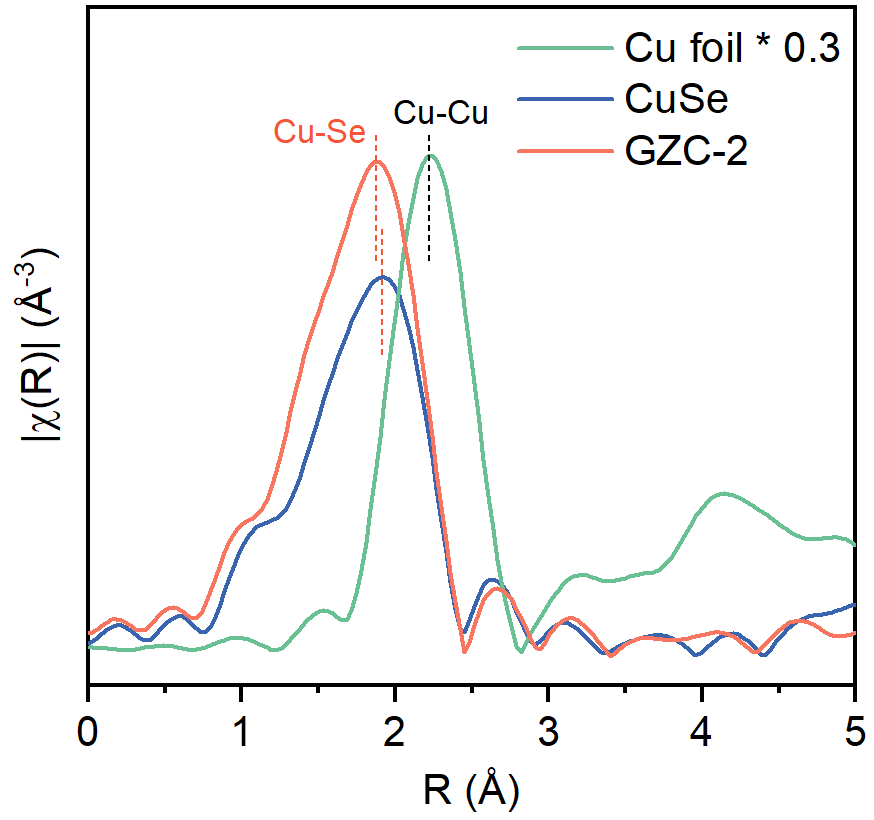


**Figure S8**. Fourier-transformed extended X-ray absorption fine structure (FT-EXAFS) spectra for GZC-2, CuSe and Cu foil.


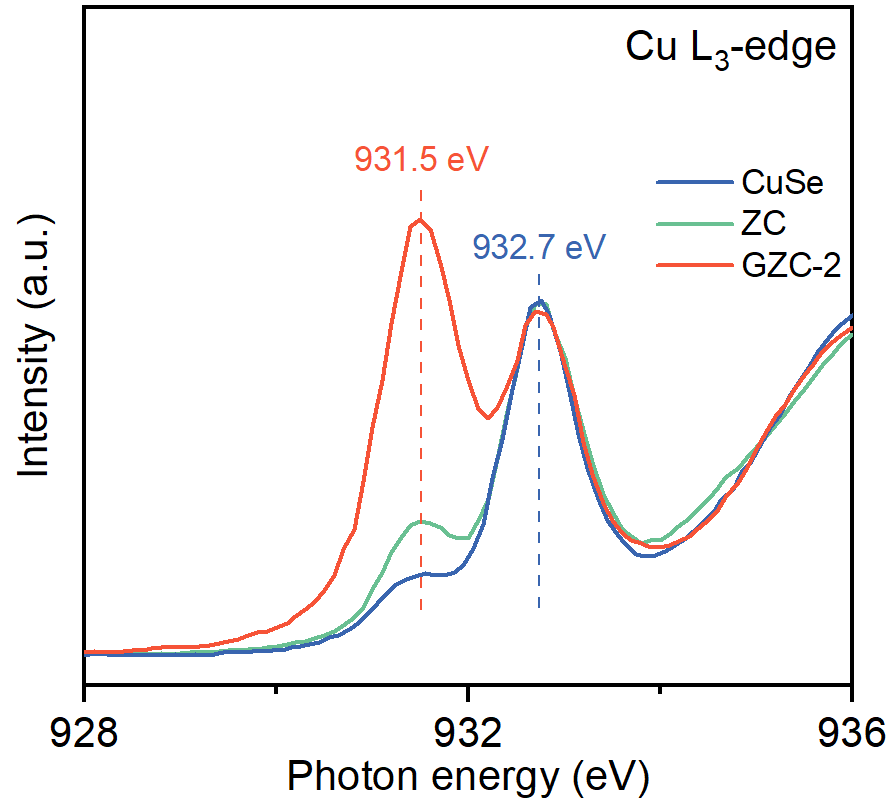


**Figure S9**. Cu L-edge XANES spectra of CuSe, ZC, and GZC-2.


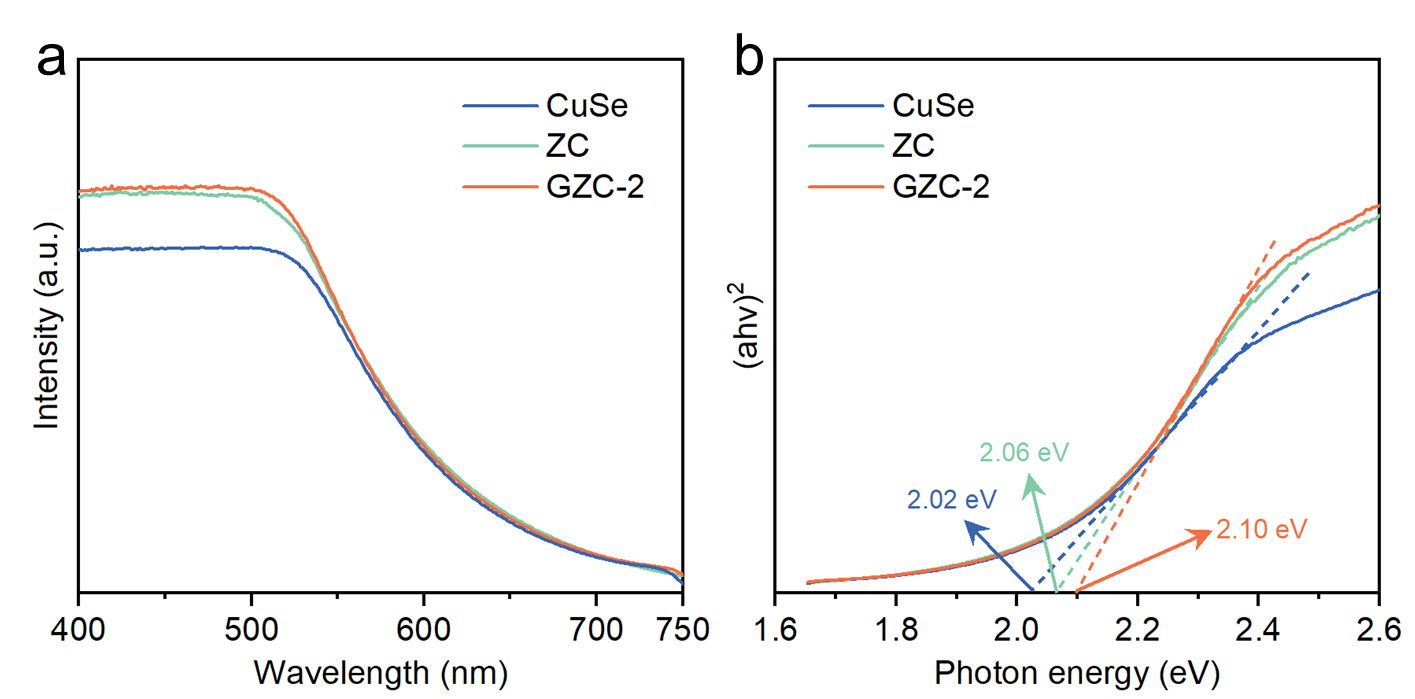


**Figure S10**. UV-Vis diffuse reflectance spectra and corresponding tauc plots of CuSe, ZC and GZC-2.


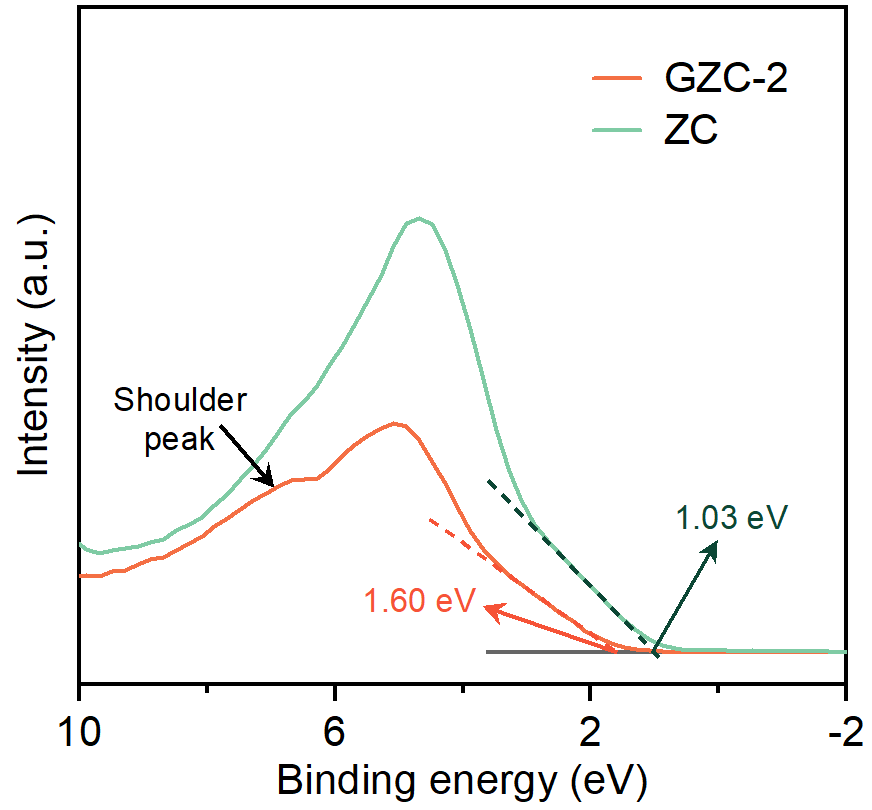


**Figure S11**. XPS-VB spectra of ZC and GZC-2.

**
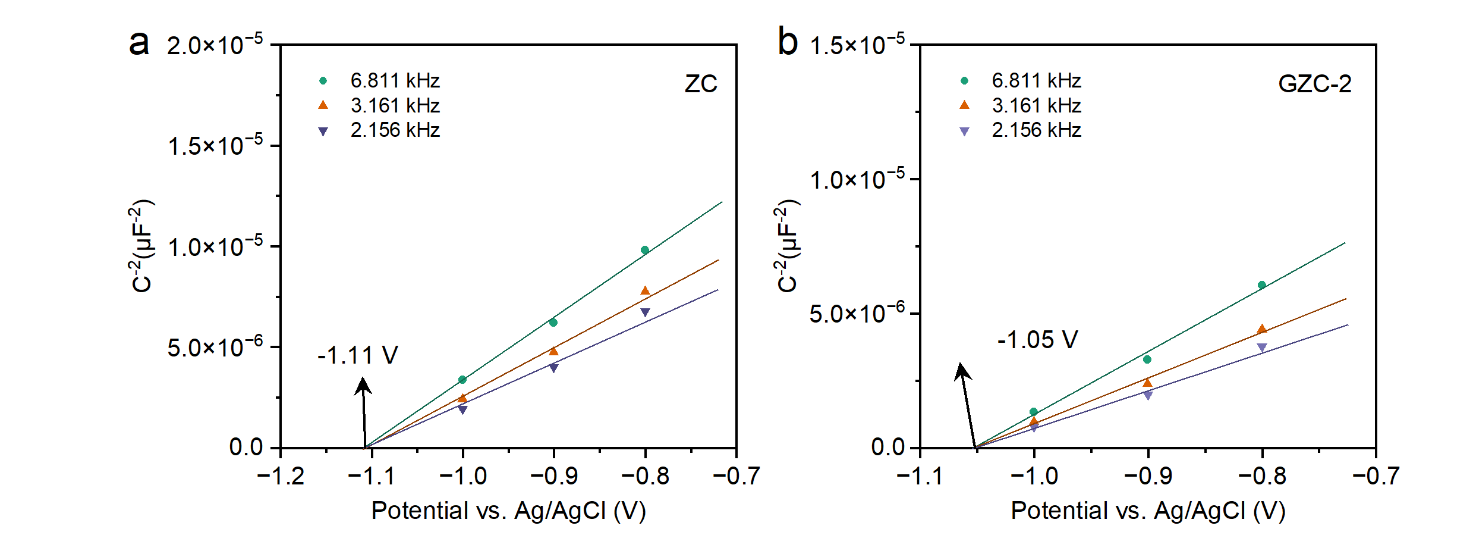
**

**Figure S12**. M-S plots of (a) ZC and (b) GZC-2.


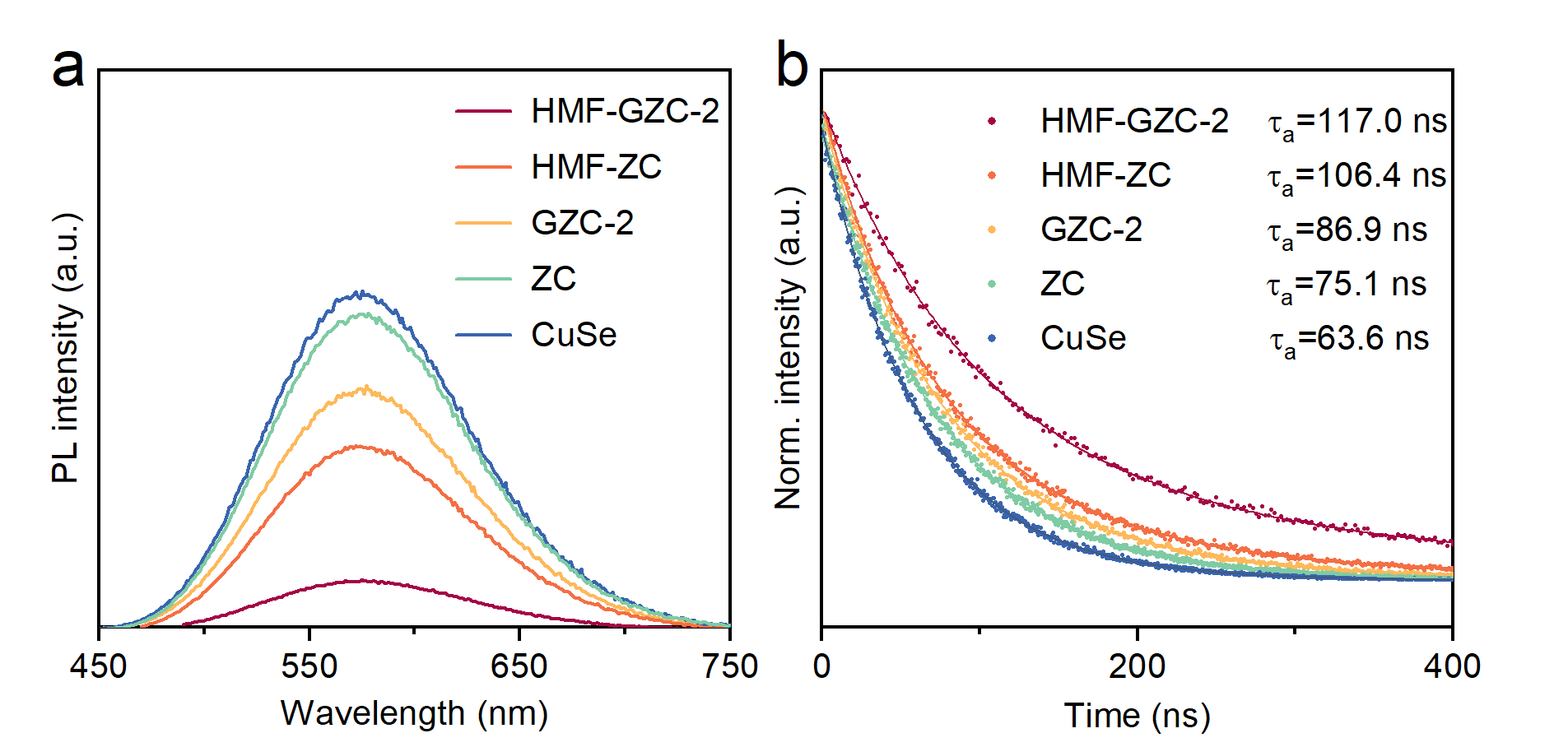


**Figure S13**. (a) Steady-state and (b) transient-state PL spectra of CuSe, ZC, GZC-2, HMF soaked GZC-2 (HMF-GZC-2) and HMF soaked ZC (HMF-ZC).


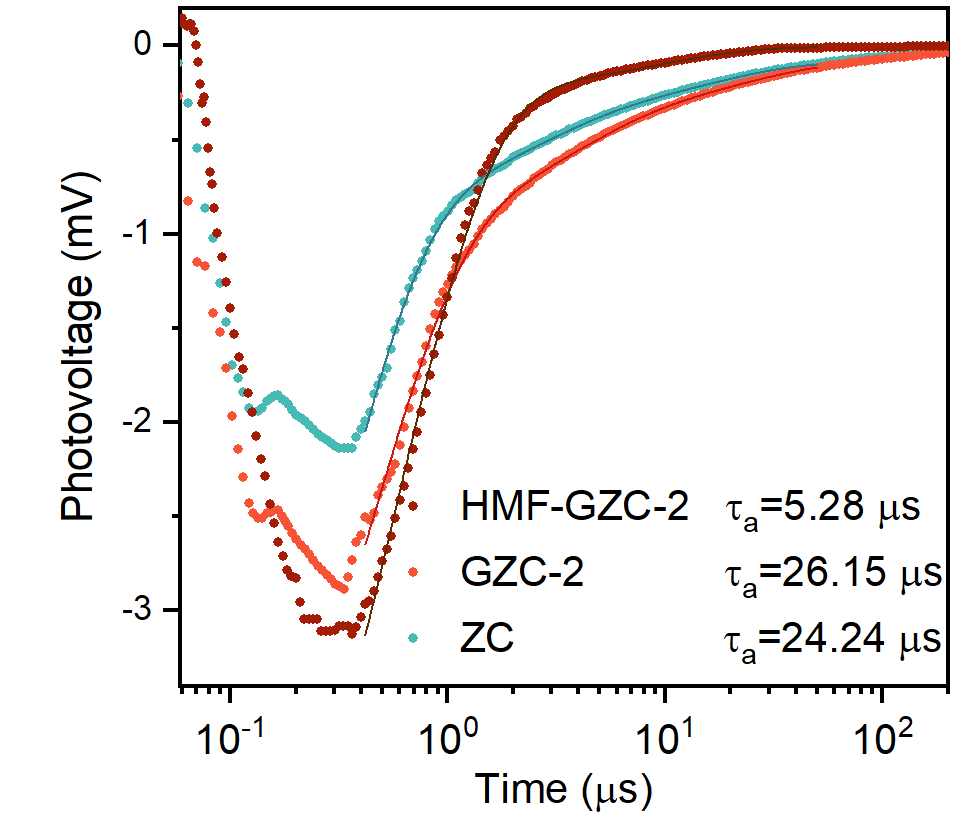


**Figure S14**. TPV spectra of ZC, GZC-2, and HMF-GZC-2.


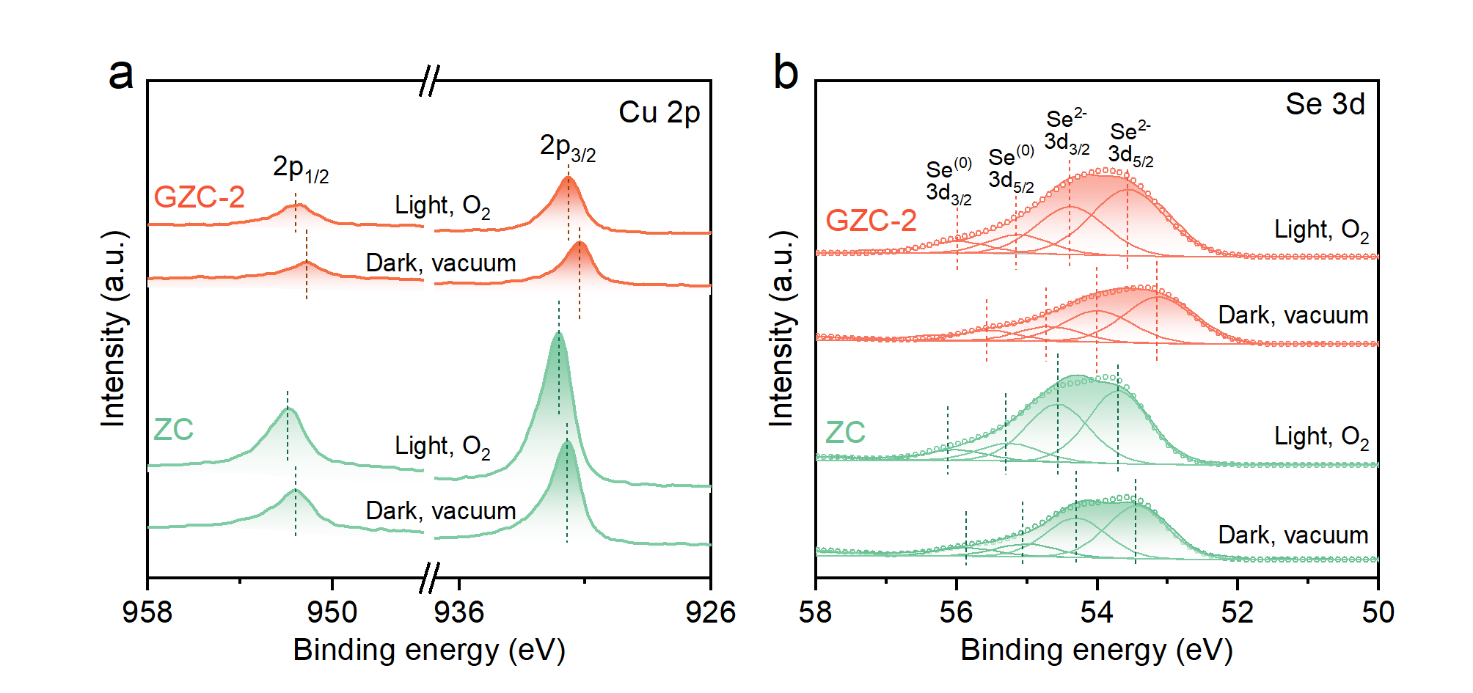


**Figure S15.** *In situ* high-Resolution XPS spectra of (a) Cu 2p and (b) Se 3d for ZC and GZC-2 in the following two conditions: i) in dark and vacuum condition; ii) with light irradiation and in O_2_ atmosphere.


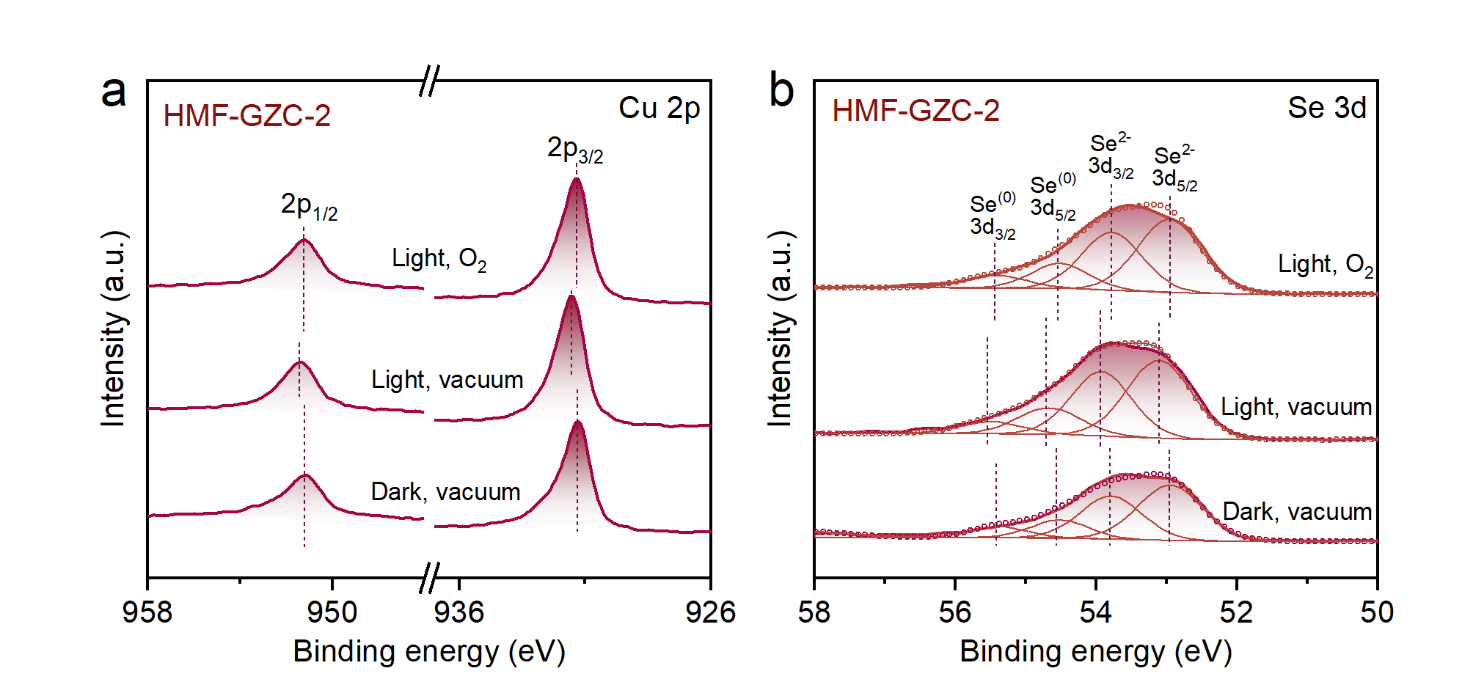


**Figure S16.** *In situ* high-Resolution XPS spectra of (a) Cu 2p and (b) Se 3d for HMF soaked GZC-2 (HMF-GZC-2) in the following three conditions: i) in dark and vacuum condition; ii) with light irradiation and in vacuum condition; iii) with light irradiation and in O_2_ atmosphere.


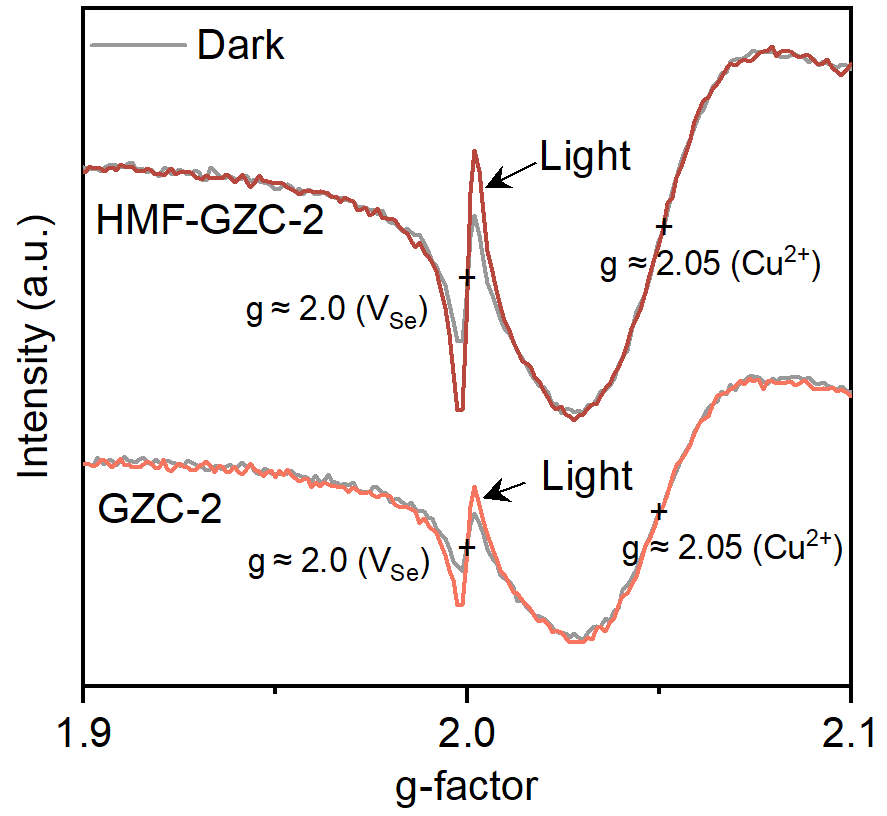


**Figure S17**. *In situ* EPR spectra of GZC-2 and HMF soaked GZC-2 (HMF-GZC-2) in the dark and under light irradiation.


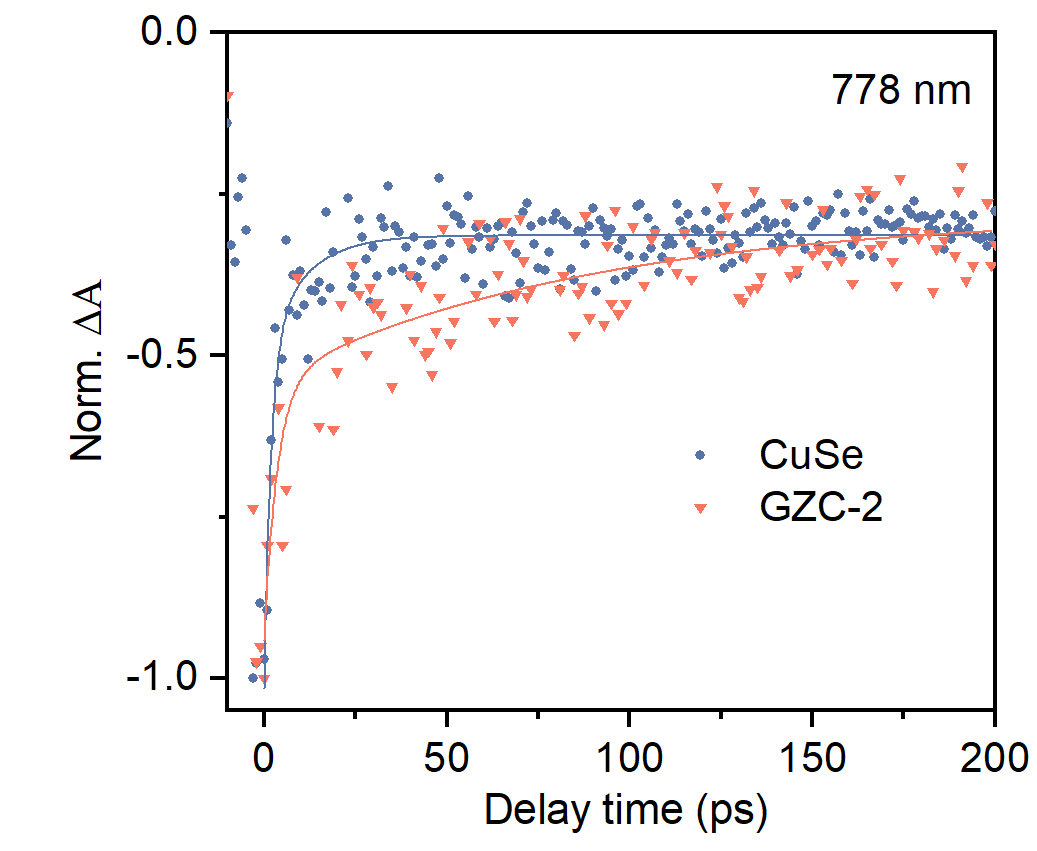


**Figure S18**. Normalized decay kinetics and fitting lines taken through the GSB peaks at ~778 nm for CuSe and GZC-2.


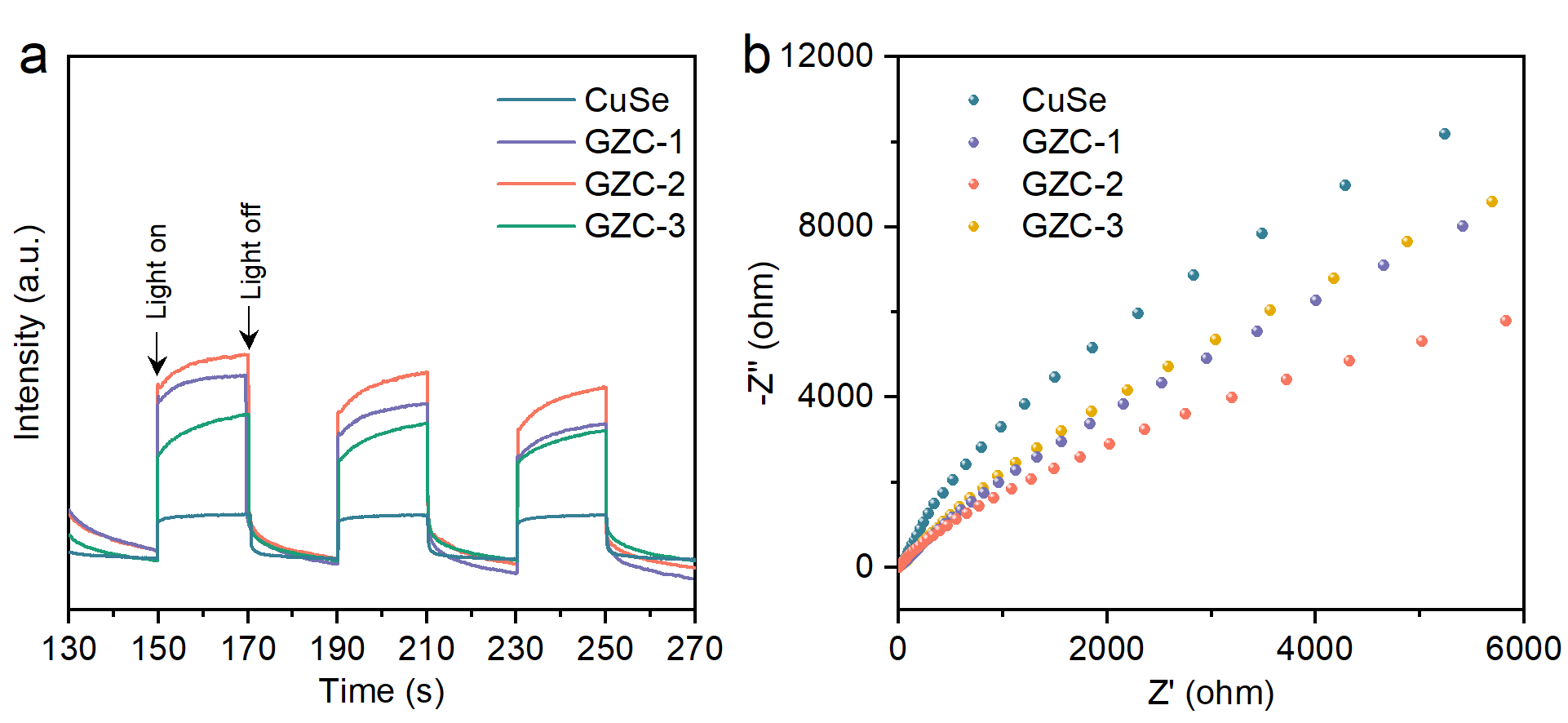


**Figure S19**. (a) Photocurrent response plots and (b) Electrochemical impedance spectroscopy plots of CuSe, GZC-1, GZC-2 and GZC-3.


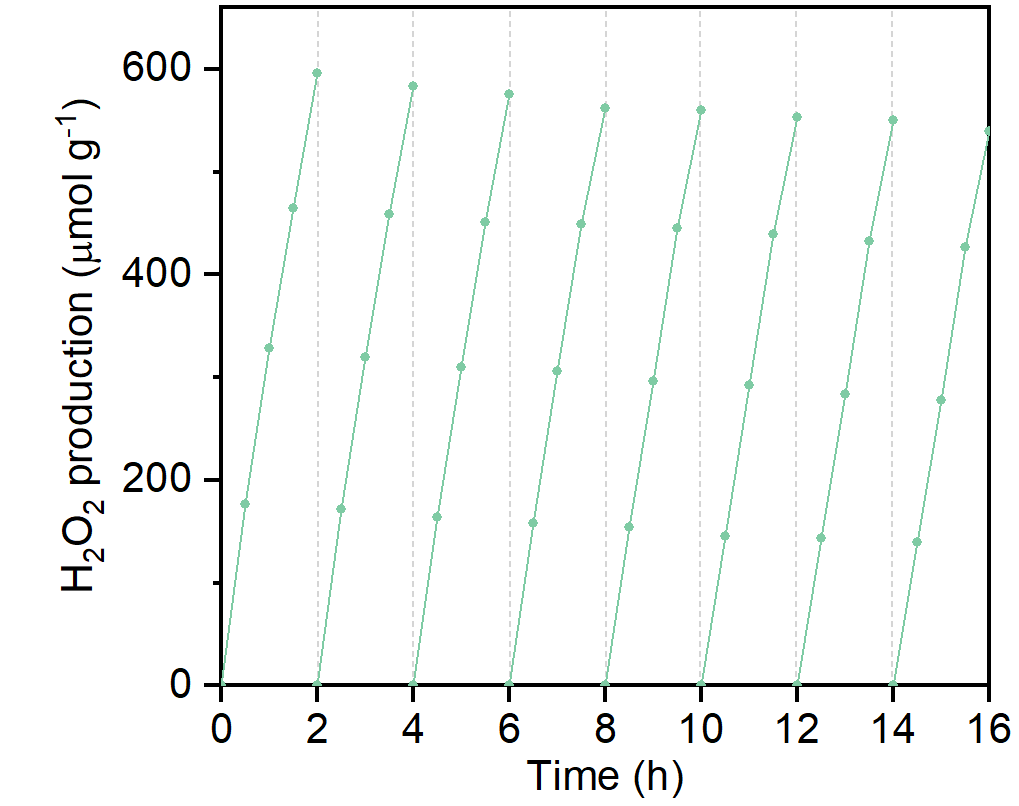


**Figure S20**. Cycling stability of GZC-2 for multiple photocatalytic runs.


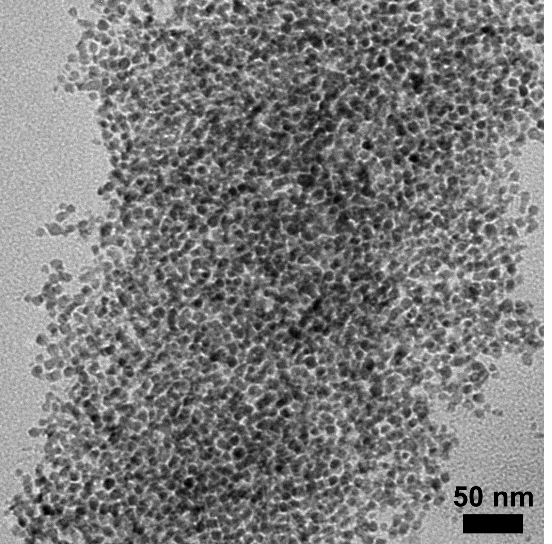


**Figure S21**. TEM image of GZC-2 after 16-h photocatalytic reaction.


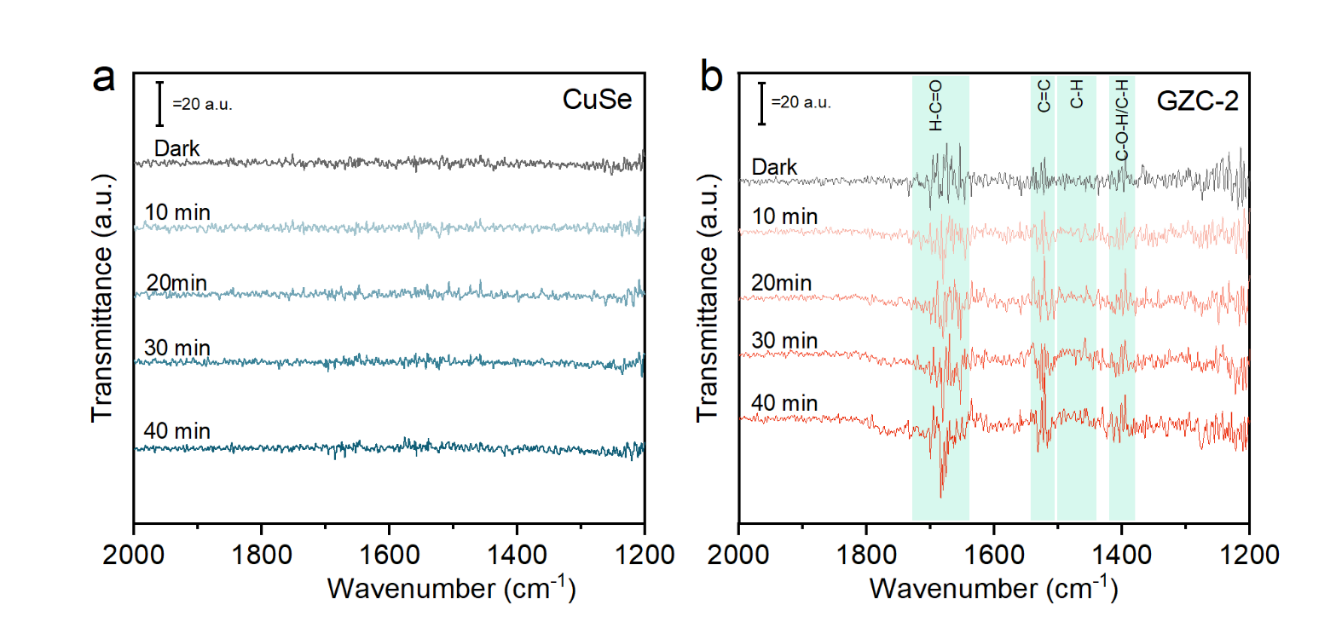


**Figure S22**. *In situ* IR spectra of (a) HMF-CuSe and (b) HMF-GZC-2 with purging of both O_2_ gas and water vapor under light irradiation.


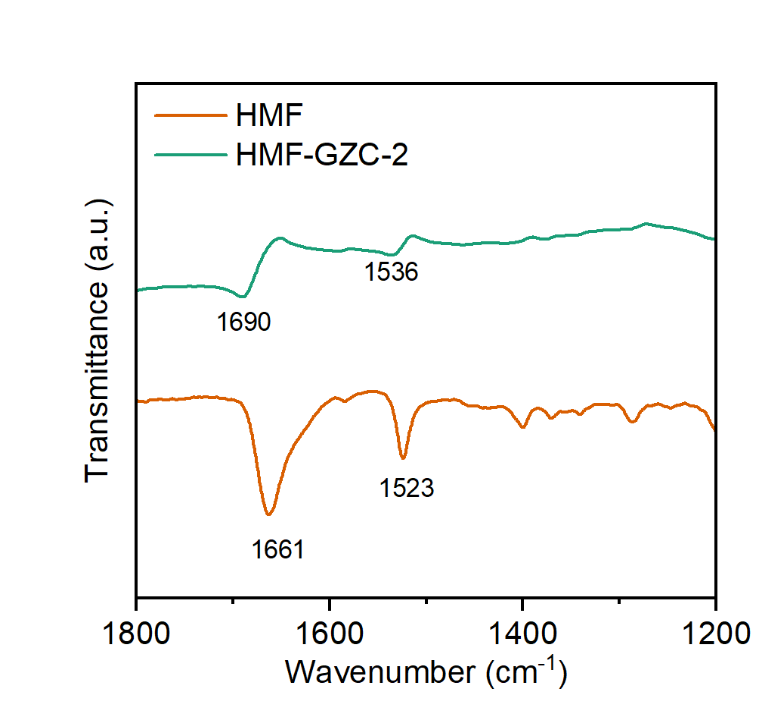


**Figure S23.** ATR-FTIR spectra results of HMF-GZC-2 and an aqueous HMF aqueous solution

(1 mM).


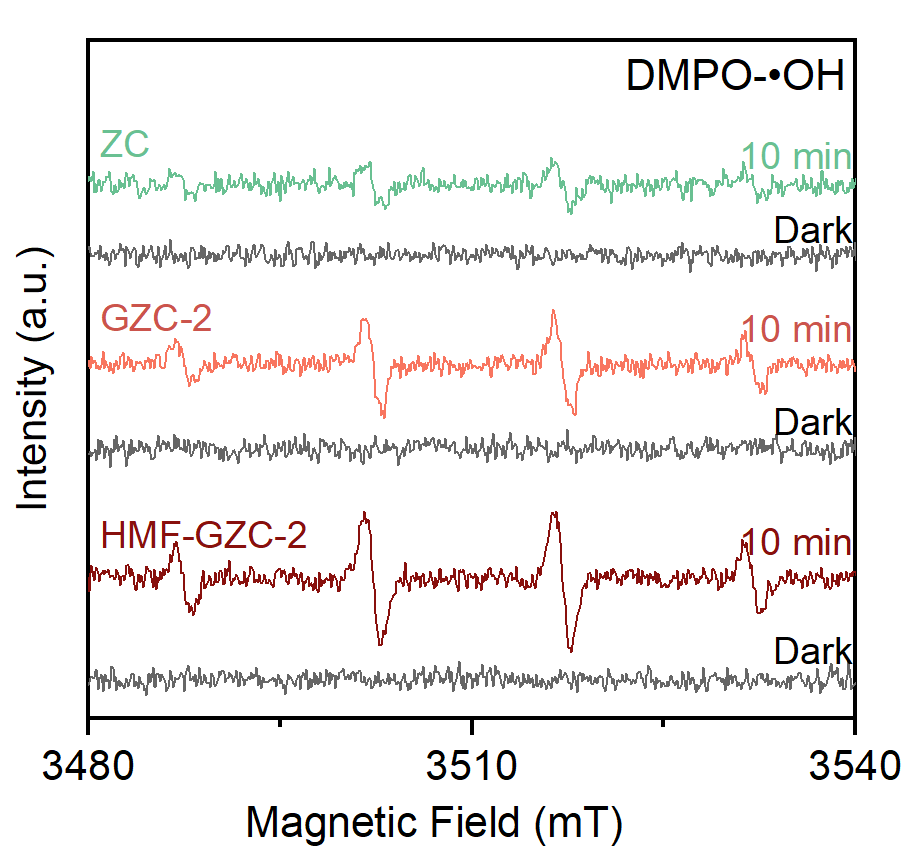


**Figure S25**. *In situ* EPR spectra of DMPO-∙OH for ZC, GZC-2 and HMF-GZC-2.

**Table S1**. Atomic ratios of Ga:Zn:Cu in GZC-1, GZC-2 and GZC-3 QDs.

| catalyst | GZC-1 | GZC-2 | GZC-3 |
| --- | --- | --- | --- |
| Atomic Ratio (Ga:Zn:Cu) | 0.0068:0.1342:1 | 0.0120:0.3553:1 | 0.0235:0.8470:1 |

**Table S2.** Reported interpretation of Cu L_3_-edge XANES peaks around 931.5 and 932.7 eV.

| Catalyst | Photon Energy (eV) | Transition | Ref. |
| --- | --- | --- | --- |
| YBa_2_Cu_3_O_6+δ_ | 931.5 | 2p_3/2_ → 3d^9^ main absorption feature (Cu^2+^) | [1] |
| Cu-MFU-4l | 931.5 | 2p_3/2_ → 3d^9^ main absorption feature (Cu^2+^) | [2] |
| CuO | 931.5 | 2p_3/2_ → 3d^9^ main absorption feature (Cu^2+^) | [3] |
| TI_2_Ba_2_Ca_2_Cu_3_O_10-δ_ | 932.8 | 2p_3/2_ → 3d^10^L transition from Cu^2+^ with ligand hole (3d^9^L) ground state | [4] |
| (Pb_2/3_Cu_1/3_)Sr_2_(Y,Ca)Cu_2_O_8+z_ | 933.0 | 2p_3/2_ → 3d^10^L transition from Cu^2+^ with ligand hole (3d^9^L) ground state | [5] |
| GZC-2 | 931.5 | 2p_3/2_ → 3d^9^ main absorption feature (Cu^2+^) | This work |
| GZC-2 | 932.7 | 2p_3/2_ → 3d^10^L transition from a Cu^2+^-Se covalent environment | This work |

**Table S3.** Band edge positions of ZC and GZC-2.

| **Samples** | **E_g_** | **E_VB_** | **E_CB_** | **E_F_**  **(vs Ag/AgCl)** | **E_F_**  **(vs RHE)** |
| --- | --- | --- | --- | --- | --- |
| ZC | 2.06 eV | 0.53 V | -1.53 V | -1.11 V | -0.5 V |
| GZC-2 | 2.10 eV | 1.16 V | -0.94 V | -1.05 V | -0.44 V |

**Table S4**. Fitting parameters for transient-state PL spectra.

| **Samples** | **τ_1_ (ns)** | **A_1_** **(%)** | **τ_2_ (ns)** | **A_2_ (%)** | **τ_a_ (ns)** |
| --- | --- | --- | --- | --- | --- |
| CuSe | 56.7 | 45.0 | 69.3 | 55.0 | 63.6 |
| ZC | 57.9 | 30.7 | 82.6 | 69.4 | 75.1 |
| GZC-2 | 65.3 | 56.4 | 114.8 | 43.6 | 86.9 |
| HMF-ZC | 81.2 | 45.0 | 99.2 | 55.0 | 91.1 |
| HMF-GZC-2 | 94.5 | 88.6 | 291.7 | 11.4 | 117.0 |

**Table S5**. Fitting parameters of TPV spectra.

| **Samples** | **τ_1_ (μs)** | **A_1_** **(%)** | **τ_2_ (μs)** | **A_2_ (%)** | **τ_3_ (μs)** | **A_3_ (%)** | **τ_a_ (μs)** |
| --- | --- | --- | --- | --- | --- | --- | --- |
| ZC | 3.46 | 16.04 | 3.34 | 11.26 | 32.53 | 72.70 | 24.24 |
| GZC-2 | 4.49 | 18.62 | 4.89 | 13.44 | 37.17 | 67.94 | 26.15 |
| HMF-GZC-2 | 6.40 | 29.42 | 6.40 | 26.01 | 11.05 | 44.58 | 5.28 |

**Table S6.** The binding energies (eV) of XPS peaks for Zn, Cu, (Ga) and Se elements in ZC, GZC-2, and HMF-GZC-2 QDs under various conditions.

| **Catalysts** | **Conditions** | **Zn 2p_1/2_** | **Zn 2p_3/2_** | **Cu 2p_1/2_** | **Cu 2p_3/2_** | **Ga 2p_3/2_** | **Se^0^ 3d_3/2_** | **Se^0^ 3d_5/2_** | **Se^2-^ 3d_3/2_** | **Se^2-^ 3d_5/2_** |
| --- | --- | --- | --- | --- | --- | --- | --- | --- | --- | --- |
| ZC | Dark, vacuum | 1044.37 | 1021.24 | 951.59 | 931.71 | --- | 55.89 | 55.04 | 54.31 | 53.44 |
| ZC | Light, O_2_ | 1044.79 | 1021.66 | 951.92 | 932.04 | --- | 56.14 | 55.29 | 54.56 | 53.69 |
| GZC-2 | Dark, vacuum | 1044.13 | 1021.01 | 951.12 | 931.24 | 1117.06 | 55.57 | 54.72 | 54.01 | 53.15 |
| GZC-2 | Light, O_2_ | 1044.37 | 1021.25 | 951.57 | 931.69 | 1117.45 | 55.98 | 55.13 | 54.42 | 53.56 |
| HMF-GZC-2 | Dark, vacuum | 1043.97 | 1020.87 | 951.22 | 931.32 | 1116.95 | 55.41 | 54.56 | 53.79 | 52.95 |
| HMF-GZC-2 | Light, vacuum | 1044.09 | 1020.99 | 951.44 | 931.54 | 1117.14 | 55.56 | 54.71 | 53.94 | 53.10 |
| HMF-GZC-2 | Light, O_2_ | 1043.98 | 1020.88 | 951.23 | 931.33 | 1117.00 | 55.43 | 54.58 | 53.81 | 52.97 |

**Table S7**. Fitting parameters of TAS spectra.

| **Samples** | **Probe wavelength** | **τ_1_ (ps)** | **A_1_** (%) | **τ_2_ (ps)** | **A_2_** (%) |
| --- | --- | --- | --- | --- | --- |
| CuSe | 788 nm | 2.32 | 21.84 | 76.31 | 78.16 |
| CuSe | 778 nm | 1.90 | 34.21 | 10.36 | 65.79 |
| GZC-2 | 788 nm | 4.67 | 12.95 | 53.74 | 87.05 |
| GZC-2 | 778 nm | 3.65 | 6.04 | 87.87 | 93.96 |

**Table S8.** Current advances for photocatalytic selective HMF-to-DFF conversion.

| **No.** | **Photocatalyst** | **Catalyst amount (mg)** | **Light Source** | **Condition** | **Reaction time (h)** | **Conv. (%)** | **Sel. (%)** | **Ref.** |
| --- | --- | --- | --- | --- | --- | --- | --- | --- |
| 1 | 12% Bi_2_WO_6_/mpg-C_3_N_4_ | 50 | 300 W xenon lamp | HMF (0.1 M, 50 mL) | 6 | 59.3 | 84.3 | [6] |
| 2 | 12.5% MoS_2_/CdIn_2_S_4_ | 10 | 7 W LED lamp (>420 nm) | HMF (0.5 mM, 10 mL) | 5 | 61.73 | 80.93 | [7] |
| 3 | 4.7% WO_3_/g-C_3_N_4_ | 50 | 300 W xenon lamp | HMF (0.1 M, 5 mL) | 6 | 27.4 | 87.2 | [8] |
| 4 | g-C_3_N_4_ | 25 | xenon lamp (λ>400 nm) | HMF (0.1 mM, 50 mL) | 4 | 26.6 | 35.6 | [9] |
| 5 | S-g-C_3_N_4_ | 25 | xenon lamp (λ>400 nm) | HMF (0.1 mM, 50 mL) | 4 | 20.1 | 28.4 | [9] |
| 6 | 20% CoP/Zn_0.5_Cd_0.5_S | 100 | 300 W xenon lamp (λ>420 nm) | HMF (0.1 M, 50 mL) | 8 | --- | 87 | [10] |
| 7 | TiO_2_@UIO-67-Zr/Ti | 10 | 300 W xenon lamp | HMF (150 mM, 2 mL) | 5 | 94 | 70 | [11] |
| 8 | V_2_O_5_/g-C_3_N_4_ | 100 | 300 W xenon lamp | HMF (20 mM, 10 mL) | 6 | 66.7 | 65.4 | [12] |
| 9 | Pt/ZIS/MnO_2_ | 30 | 300 W xenon lamp (λ>400 nm) | HMF (20 mM, 10 mL) | 6 | --- | 85.4 | [13] |
| 10 | P25 | 20 | visible light (λ=515 nm) | HMF (1 mM, 20 mL) | 4 | 18 | 69 | [14] |
| 11 | SGH-TiO_2_ | 20 | visible light (λ=515 nm) | HMF (1 mM, 20 mL) | 4 | 59 | 87 | [14] |
| 12 | MAPbBr_3_ | 40 | LED (λ=450 nm) | HMF (5 mM, 10 mL) | 10 | 100 | 90 | [15] |
| 13 | Cu SAs/p-CNS | 5 | 20 W blue LED (λ=455 nm) | HMF (10 mM, 5mL) | 4 | 77.1 | 85.6 | [16] |
| 14 | Cd_1.5_In_2_S_4.5_ | 20 | 500 W Xenon lamp (>420 nm) | HMF (4 mg/mL, 5 mL) | 6 | 68.8 | 62.7 | [17] |
| 15 | Bi_2_WO_6_ | 20 | 300 W Xenon lamp (>420 nm) | HMF (4 mg/mL, 5 mL) | 10 | 25.1 | 73.4 | [18] |
| 16 | N-TiO_2_ | 100 | 500 W lamp (365 nm) | HMF (0.5 mM, 20 mL) | 4 | 60 | 30 | [19] |
| 17 | Amorphous TiO_2_ | 30 | Fluorescent lamp (365 nm) | HMF (0.5 mM, 150 mL) | 4 | 20 | 22 | [20] |
| 18 | **GSC-2** | 10 | 300 W xenon lamp | HMF (0.5 mM, 20 mL) | 4 | 89 | 91.0 | **This work** |

**Table S9.** Reported FTIR peak assignments around 1683, 1520, 1475, and 1400 cm^-1^.

| Wavenumber (cm^-1^) | Assignment | Ref. |
| --- | --- | --- |
| 1670 | C=O stretching | [21] |
| 1635 | C=O stretching | [22] |
| 1672 | C=O stretching | [23] |
| 1520 | C=C stretching | [23] |
| 1700 | C=O stretching | [24] |
| 1443 | deformation and symmetrical stretching vibration of C-H bond in CH_2_OH group | [24] |
| 1465 | scissoring mode of the CH_2_ group | [25] |
| 1350-1400 | aliphatic C-H scissoring/symmetric deformation | [26] |
| 1370-1400 | non-carboxyl C-O-H in-plane bend and CH_2_ deformation | [27] |
| 1683 | C=O stretching | This work |
| 1520 | conjugated C=C stretching | This work |
| 1475 | C-H in-plane bending | This work |
| 1400 | C-O-H bending modes of HMF/the C-H wagging or scissoring modes of HMF/DFF | This work |

**Reference:**

[1] N. Gauquelin, D.G. Hawthorn, G.A. Sawatzky, R.X. Liang, D.A. Bonn, W.N. Hardy, G.A. Botton, Atomic scale real-space mapping of holes in YBa_2_Cu_3_O_6+δ_, *Nat. Commun.* **2014**, 5, 4275.

[2] G.M. Su, H. Wang, B.R. Barnett, J.R. Long, D. Prendergast, W.S. Drisdell, Backbonding contributions to small molecule chemisorption in a metal-organic framework with open copper(i) centers, *Chem. Sci.* **2020**, 12, 2156-2164.

[3] H. Wang, G.M. Su, B.R. Barnett, W.S. Drisdell, J.R. Long, D. Prendergast, Understanding 2p core-level excitons of late transition metals by analysis of mixed-valence copper in a metal-organic framework, *Phys. Chem. Chem. Phys.* **2024**, 26, 11980-11987.

[4] J.M. Chen, S.C. Chung, High-resolution X-ray absorption near edge structure studies of monophasic TI_2_Ba_2_Ca_2_Cu_3_O_10-δ_ superconductor, *Solid State Commun.* **1996**, 99, 493-498.

[5] M. Karppinen, M. Kotiranta, H. Yamauchi, P. Nachimuthu, R.S. Liu, J.M. Chen, O K-edge and Cu L_23_-edge XANES study on the concentration and distribution of holes in the(Pb_2/3_Cu_1/3_)_3_Sr_2_(Y, Ca)Cu_2_O_8+z_ superconductive phase, *Phys. Rev. B* **2001**, 63, 184507.

[6] L. Cheng, D. Huang, Y. Zhang, Y. Wu, Photocatalytic selective oxidation of HMF to DFF over Bi_2_WO_6_/mpg-C_3_N_4_ composite under visible light, *Appl. Organomet. Chem.* **2021**, 35, e6404.

[7] Q. Zhu, Y. Zhuang, H. Zhao, P. Zhan, C. Ren, C. Su, W. Ren, J. Zhang, D. Cai, P. Qin, 2,5-Diformylfuran production by photocatalytic selective oxidation of 5-hydroxymethylfurfural in water using MoS_2_/CdIn_2_S_4_ flower-like heterojunctions, *Chin. J. Chem. Eng.* **2023**, 54, 180-191.

[8] H. Zhang, Z. Feng, Y. Zhu, Y. Wu, T. Wu, Photocatalytic selective oxidation of biomass-derived 5-hydroxymethylfurfural to 2,5-diformylfuran on WO_3_/g-C_3_N_4_ composite under irradiation of visible light, *J. Photochem. Photobiol. A Chem.* **2019**, 371, 1-9.

[9] A. Zheng, K. Li, H. Duan, H. Shi, Comparison investigation on the photocatalytic conversion of 5-hydroxymethylfurfural on g-C_3_N_4_ and S-doped g-C_3_N_4_: Performance and mechanism, *ChemistrySelect* **2024**, 9, e202405169.

[10] Y. Yang, W. Ren, X. Zheng, S. Meng, C. Cai, X. Fu, S. Chen, Decorating Zn_0.5_Cd_0.5_S with C,N Co-doped CoP: An efficient dual-functional photocatalyst for H_2_ evolution and 2,5-diformylfuran oxidation, *ACS Appl. Mater. Interfaces* **2022**, 14, 54649-54661.

[11] Y. Zhou, J. Liu, J. Long, Photocatalytic oxidation 5-hydroxymethylfurfural to 2,5-diformylfuran under air condition over porous TiO_2_@MOF, *J. Solid State Chem.* **2021**, 303, 122510.

[12] Y. Lin, D. Huang, C. Gong, Y. Zhou, Y. Wu, Simple synthesis of V_2_O_5_/g-C_3_N_4_ photocatalyst for the oxidation of biomass-derived 5-hydroxymethylfurfural, *Res. Chem. Intermed.* **2025**, 51, 1473-1489.

[13] Z. Yang, X. Xia, M. Fang, L. Wang, Y. Liu, Photothermal effect act as controllable switch for tunable photocatalytic selective oxidation of 5-hydroxymethylfurfural, *Chem. Eng. J.* **2023**, 476, 146544.

[14] A. Khan, M. Goepel, A. Kubas, D. Lomot, W. Lisowski, D. Lisovytskiy, A. Nowicka, J.C. Colmenares, R. Glaser, Selective oxidation of 5-hydroxymethylfurfural to 2,5-diformylfuran by visible light-driven photocatalysis over in situ substrate-sensitized titania, *ChemSusChem* **2021**, 14, 1351-1362.

[15] M. Zhang, Z. Li, X. Xin, J. Zhang, Y. Feng, H. Lv, Selective valorization of 5-hydroxymethylfurfural to 2,5-diformylfuran using atmospheric O_2_ and MAPbBr_3_ perovskite under visible light, *ACS Catalysis* **2020**, 10, 14793-14800.

[16] G. Wang, R. Huang, J. Zhang, J. Mao, D. Wang, Y. Li, Synergistic modulation of the separation of photo-generated carries via engineering of dual atomic sites for promoting photocatalytic performance, *Adv. Mater.* **2021**, 33, 2105904.

[17] M. Zhang, Z. Yu, J. Xiong, R. Zhang, X. Liu, X. Lu, One-step hydrothermal synthesis of Cd_x_In_y_S_(x+1.5y)_ for photocatalytic oxidation of biomass-derived 5-hydroxymethylfurfural to 2, 5-diformyl-furan under ambient conditions, *Appl. Catal. B Environ.* **2022**, 300, 120738.

[18] A. Kumar, R. Srivastava, Rose-like Bi_2_WO_6_ nanostructure for visible-light-assisted oxidation of lignocellulose-derived 5-hydroxymethylfurfural and vanillyl alcohol, *ACS Appl. Nano Mater.* **2021**, 4, 9080-9093.

[19] I. Krivtsov, M. Ilkaeva, E. Salas-Colera, Z. Amghouz, J.R. García, E. Díaz, S. Ordóñez, S. Villar-Rodil, Consequences of nitrogen doping and oxygen enrichment on titanium local order and photocatalytic performance of Tio_2_ anatase, *J. Phys. Chem. C* **2017**, 121, 6770-6780.

[20] S. Yurdakal, B.S. Tek, O. Alagöz, V. Augugliaro, V. Loddo, G. Palmisano, L. Palmisano, Photocatalytic selective oxidation of 5-(hydroxymethyl)-2-furaldehyde to 2,5-furandicarbaldehyde in water by using anatase, rutile, and brookite TiO_2_ nanoparticles, *ACS Sustain. Chem. Eng.* **2013**, 1, 456-461.

[21] Y. Xie, Z. Zhou, N. Yang, G. Zhao, An overall reaction integrated with highly selective oxidation of 5‐hydroxymethylfurfural and efficient hydrogen evolution, *Adv. Funct. Mater.* **2021**, 31, 2102886.

[22] S. Fan, B. Zhu, X. Yu, Y. Gao, W. Xie, Y. Yang, J. Zhang, C. Chen, Uncovering the electrooxidation behavior of 5-hydroxymethylfurfural on Ni/Co electrodes, *J. Energy Chem.* **2024**, 92, 1-7.

[23] L. Wang, H. Jin, Z. Liu, S. Yang, G. He, H. Liu, X. Meng, C. Xu, Investigation on the preparation of 5-Hydroxymethylfurfural through fructose dehydration using in-line FTIR and in-situ 13C NMR, *J. Catal.* **2024**, 432, 115450.

[24] W. Xue, J. Ye, Z. Zhu, R. Kumar, J. Zhao, Harnessing trace water for enhanced photocatalytic oxidation of biomass-derived alcohols to aldehydes, *Energy Environ. Sci.* **2025**, 18, 214-226.

[25] D. Lin-Vien, N.B. Colthup, W.G. Fateley, J.G. Grasselli, *The handbook of infrared and raman characteristic frequencies of organic molecules*, Academic Press, San Diego, **1991**, pp. 9-28.

[26] M.C. Sánchez-Lemus, F.F. Schoeggl, S.D. Taylor, S.I. Andersen, M.M. Mapolelo, S.C. Mahavadi, H.W. Yarranton, Characterization of heavy distillation cuts using fourier transform infrared spectrometry: proof of concept, *Energ. Fuel* **2016**, 30, 10187-10199.

[27] D.S. Volkov, P.K. Krivoshein, M.A. Proskurnin, Detonation nanodiamonds: A comparison study by photoacoustic, diffuse reflectance, and attenuated total reflection FTIR spectroscopies, *Nanomaterials (Basel)* **2020**, 10, 2501.
